# General practice antibiotic prescriptions attributable to respiratory syncytial virus by age and antibiotic class: an ecological analysis of the English population

**DOI:** 10.1093/jac/dkaf043

**Published:** 2025-02-19

**Authors:** Lucy Miller, Thomas Beaney, Russell Hope, Mark Cunningham, Julie V Robotham, Koen B Pouwels, Cèire E Costelloe

**Affiliations:** Global Digital Health Unit, School of Public Health, Imperial College London, London W12 0BZ, UK; Healthcare Associated Infection, Fungal, Antimicrobial Resistance, Antimicrobial Usage and Sepsis Division, UK Health Security Agency, London NW9 5EQ, UK; Global Digital Health Unit, School of Public Health, Imperial College London, London W12 0BZ, UK; Healthcare Associated Infection, Fungal, Antimicrobial Resistance, Antimicrobial Usage and Sepsis Division, UK Health Security Agency, London NW9 5EQ, UK; Department of Primary Care and Public Health, School of Public Health, Imperial College London, London W12 0BZ, UK; Healthcare Associated Infection, Fungal, Antimicrobial Resistance, Antimicrobial Usage and Sepsis Division, UK Health Security Agency, London NW9 5EQ, UK; The National Institute for Health Research (NIHR) Health Protection Research Unit in Healthcare Associated Infections and Antimicrobial Resistance at University of Oxford, Oxford, UK; The National Institute for Health Research (NIHR) Health Protection Research Unit in Healthcare Associated Infections and Antimicrobial Resistance at University of Oxford, Oxford, UK; Nuffield Department of Primary Care Health Sciences, University of Oxford, Oxford OX2 6GG, UK; Global Digital Health Unit, School of Public Health, Imperial College London, London W12 0BZ, UK; Health Informatics Group, Institute of Cancer Research, London SW7 3RP, UK

## Abstract

**Background:**

Respiratory syncytial virus (RSV) may contribute to a substantial volume of antibiotic prescriptions in primary care. However, data on the type of antibiotics prescribed for such infections are only available for children <5 years in the UK. Understanding the contribution of RSV to antibiotic prescribing would facilitate predicting the impact of RSV preventative measures on antibiotic use and resistance. The objective of this study was to estimate the proportion of antibiotic prescriptions in English general practice attributable to RSV by age and antibiotic class.

**Methods:**

Generalized additive models examined associations between weekly counts of general practice antibiotic prescriptions and laboratory-confirmed respiratory infections from 2015 to 2018, adjusting for temperature, practice holidays and remaining seasonal confounders. We used general practice records from the Clinical Practice Research Datalink and microbiology tests for RSV, influenza, rhinovirus, adenovirus, parainfluenza, human metapneumovirus, *Mycoplasma pneumoniae* and *Streptococcus pneumoniae* from England’s Second Generation Surveillance System.

**Results:**

An estimated 2.1% of antibiotics were attributable to RSV, equating to an average of 640 000 prescriptions annually. Of these, adults ≥75 years contributed to the greatest volume, with an annual average of 149 078 (95% credible interval: 93 733–206 045). Infants 6–23 months had the highest average annual rate at 6580 prescriptions per 100 000 individuals (95% credible interval: 4522–8651). Most RSV-attributable antibiotic prescriptions were penicillins, macrolides or tetracyclines. Adults ≥65 years had a wider range of antibiotic classes associated with RSV compared with younger age groups.

**Conclusions:**

Interventions to reduce the burden of RSV, particularly in older adults, could complement current strategies to reduce antibiotic use in England.

## Introduction

Optimizing antibiotic use by reducing unnecessary prescriptions and ensuring provision for those needing treatment are essential priorities to mitigate the significant threat antibiotic-resistant infections pose to healthcare. The UK’s 2024 National Action Plan aims to reduce total human antibiotic use by 5% by 2029 from a 2019 baseline.^1^ It has been suggested that respiratory syncytial virus (RSV) may generate a considerable number of primary care antibiotic prescriptions in the UK,^2–4^ most of which are anticipated to be unnecessary, given RSV presentations in primary care are typically self-limiting.^5^

Several RSV prophylactics, including vaccines and monoclonal antibodies (mABs) targeting infants, pregnant women and older adults, have been licensed in the UK.^6^ These interventions may considerably reduce the burden of RSV and subsequent antibiotic use, which could impact resistant infections downstream.^6,7^ A secondary analysis of a trial indicated that a maternal vaccine could prevent 3.9 courses of antibiotics per 100 infants during their first year of life.^8^ However, vaccines for older adults in the UK may offer a greater impact, as this group has the highest rates of general practice (GP) antibiotic prescriptions^9^ and a considerable health burden from RSV.^3,10^

Understanding which population groups’ antibiotic prescribing is most likely affected by these programmes is important for informing their implementation and strategies to reduce antimicrobial resistance (AMR). Models that can predict the impact of programmes on antibiotic prescribing and subsequent resistant infections, incorporating the specific types of antibiotics likely to be reduced, are needed, as it is well established that antibiotics vary in their selection for resistance.^11,12^ However, evidence of RSV-attributable antibiotic prescribing described by antibiotic type is only available for children <5 years in the UK.^4^

In this ecological study, we aimed to estimate the proportion of antibiotic prescriptions in English GPs attributable to RSV by age and antibiotic class.

## Methods

### Ethics

The study protocol was approved by the Clinical Practice Research Datalink (CPRD) Independent Scientific Advisory Committee (protocol 20_000283) and the Imperial College Research Ethics Committee (reference number 21IC6607).

### Study period and data

We used separate age-specific ecological regression analyses to estimate weekly antibiotic prescriptions attributable to RSV from 29 December 2014 to 30 December 2018, using weekly counts of laboratory-confirmed respiratory infections, average weekly temperatures and indicator variables for practice holiday weeks as explanatory covariates. The study period was selected to avoid the impact of the COVID-19 pandemic on antibiotic prescribing and respiratory infections.^13–15^

Data on antibiotic prescriptions were obtained from CPRD Aurum, a dataset containing anonymized electronic health records from contributing GPs in England. This dataset represents ∼23% of the English population^16,17^ and broadly reflects national demographics regarding age, sex and deprivation.^18^ Records of research-acceptable patients registered between 1 January 2015 and 1 January 2020 and linked to hospital records from the Hospital Episode Statistics (HES) database and practice-level Index of Multiple Deprivation (IMD) were extracted.^19^ CPRD determines research acceptability based on data reliability, including date of birth, practice registration date and transfer out date.^20^ The study period began 3 days before the extraction period to align antibiotic prescriptions with calendar weeks, ensuring consistent weekly counts by evenly distributing weekends when most practices were closed. This slight misalignment excluded individuals registered only during those 3 days and their subsequent prescriptions. However, the exclusion was negligible, representing <0.0006% of antibiotic prescriptions if periods were aligned. Linkage to HES and IMD, required for analyses outlined in the CPRD protocol, resulted in the exclusion of approximately 2.5% of research-acceptable patients.

Antibiotic prescriptions were identified irrespective of the presenting condition, as up to a third could have missing or non-specific diagnostic codes in English primary care.^21^ Antibiotics included any systemic antibacterial (J01) listed in the Anatomical Therapeutic Chemical classification system^22^ and Chapter 5.1 of the British National Formulary (BNF),^23^ excluding those used for leprosy, tuberculosis and topical applications, except for those recommended for ear infections.^24^ The primary outcomes included respiratory antibiotics primarily used for respiratory tract infections (RTIs),^21,24–29^ namely amoxicillin, phenoxymethylpenicillin, clarithromycin, erythromycin and doxycycline, along with antibiotic class groups defined by the BNF Chapter 5.1 subsections.^23^ The secondary outcomes included total antibiotic prescriptions and respiratory antibiotics of potential importance for resistance to assess RSV’s contribution to overall antibiotic use across different age groups and the resistance propensity of these prescriptions. Respiratory antibiotics were defined more conservatively than those potentially important for resistance, including only those with minimal non-respiratory use to better represent respiratory prescribing in the absence of diagnostic codes for RTI presentations. Full outcome definitions are provided in Section 1.1 and Table S1 of the supplementary data (available as Supplementary data at *JAC* Online). Nitrofurantoin prescriptions typically used only for urinary tract infections (UTIs)^21,30^ were analysed as a negative control to identify any unaccounted residual confounding. Antibiotics were not grouped when the same antibiotic was prescribed to the same patient within a defined period (e.g. 2 weeks), as we aimed to capture the total volume of prescriptions associated with infections, a key driver of resistance.^31^ Instead, all prescriptions were aggregated by calendar week and stratified by age: 0–5 and 6–23 months and 2–4, 5–14, 15–44, 45–64, 65–74 and ≥75 years.

Positive laboratory tests of respiratory pathogens in the English population, including RSV, influenza, rhinovirus, adenovirus, parainfluenza, human metapneumovirus (hMPV), *Mycoplasma pneumoniae* and *Streptococcus pneumoniae*, were extracted from the UK Health Security Agency’s Second Generation Surveillance System (SGSS). The SGSS collects routine infectious disease test results from around 98% of hospital laboratories in England.^32,33^ Tests are voluntarily submitted by healthcare professionals, with clinically significant infections likely captured and most culture requests originating from hospitals. We included all respiratory samples for viruses and respiratory and invasive samples for *M. pneumoniae*. Only invasive samples were included for *S. pneumoniae* due to inconsistent reporting of respiratory samples.^33^ Tests from the same patient for the same pathogen were grouped if reported within 2 weeks (6 weeks for influenza) to prevent over-reporting.^33^ After grouping, tests were assigned the date of the first infection report and aggregated by calendar week, representing the first week of their infection episode. Grouped tests averaged 1.85 reports per episode across all pathogens. Tests were then stratified into broad age groups (0–4, 5–64 and ≥65 years) to account for age-specific differences in infection seasonality.^34^ These broader age groups prevented low weekly infection counts that could hinder associations with prescriptions.

Daily average temperatures for England were obtained from the Hadley Centre Central England Temperature dataset^35^ and averaged by calendar week. These daily temperature records roughly represent the area between Bristol, London and Lancashire and are adjusted for urban warming.^35^ They are assumed to provide a reasonable average for England, despite regional differences.

### Statistical analysis

Separate models were developed for each outcome by age, associating outcomes with the corresponding weekly counts of laboratory-confirmed infections in broad age groups and average temperatures. We explored the seasonality of antibiotic prescriptions and laboratory-confirmed infections and used correlation matrices to examine the collinearity between pathogens and temperature.

We fitted generalized additive models (GAMs) with a negative binomial distribution and an identity link. The negative binomial distribution accounted for overdispersion in outcome counts, while the identity link ensured each laboratory-confirmed infection contributed additively to these counts. GAMs allowed for non-linear covariate relationships using splines, fitting data-derived trends using restricted maximum likelihood estimation (REML),^36^ providing flexible adjustment for unmeasured seasonal confounding. Splines were expanded to penalize covariates with no relationship for variable selection (double penalty approach), enabling penalization for deviations from a straight line and straight-line components to be shrunk to 0.^36^ To adjust for an increasing CPRD population, all covariates, including the intercept, were multiplied by the average mid-year CPRD population of the relevant age group and calendar week, effectively applying an offset (Table S2 and Equation S1). This method preserves the scale in identity link models, enabling straightforward interpretation of the results as absolute changes in outcome counts.

Positive tests, likely from hospitalized patients, were assumed to reflect community incidence, as both are expected to occur within approximately a week of each other. For instance, paediatric RSV hospitalizations are reported to occur 3–4 days after symptom onset,^37^ and the average duration of RTI symptoms is estimated to be around 3–14 days.^38,39^ Weekly lags were applied to align peaks in confirmed RSV infections in broad age groups with those used for antibiotic outcomes, ensuring that broader groups approximately reflected infections in specific age groups analysed. This alignment was evaluated using autocorrelation function (ACF) plots. To improve model fit, reductions in the Akaike information criterion were used to evaluate the inclusion of 3-week moving averages of pathogen counts to smooth irregularities, linear and non-linear outcome trends to adjust for unmeasured seasonal confounding and indicator variables flagging practice holiday weeks to account for outliers in outcome counts. Pathogen counts and temperatures were fitted with penalized splines using REML with a double penalty to explore non-linear trends and conduct variable selection.

GAMs were performed using the ‘mgcv’ package in R version 4.4.1.^40^ Model fit was also assessed by comparing posterior simulations with observed data. Posterior simulations were generated using the ‘postSim’ function from the mgcViz package,^41^ ensuring they were drawn from a multivariate normal distribution defined by the model’s variance–covariance matrix, incorporating uncertainties and correlations among covariates.^41,42^ Model stationary was assessed using ACF, residual and quantile–quantile plots. The results of model fitting are provided in Section 2.2 of supplementary data.

For each age-specific model of outcomes, RSV-attributable prescriptions were estimated by subtracting posterior simulations of weekly outcome counts from a model with RSV counts set to 0, from those of a model using the observed RSV counts. Simulations were repeated 1000 times, taking the 2.5% and 97.5% percentiles to estimate 95% credible intervals (CrI). Prescribing rates were estimated using age-specific mid-year CPRD study population estimates and scaled to the national level with age-specific Office of National Statistics (ONS) mid-year English population estimates between 2015 and 2018 (Table S2).^17^ Average age-specific counts of RSV-attributable antibiotic classes were used to estimate age-specific class proportions of RSV-attributable antibiotic prescriptions. Only class counts with an estimated 2.5% percentile above 0 were considered in this estimation, excluding UTI antibiotic prescriptions.

### Sensitivity analysis

All respiratory pathogens included in the GAMs have evidence of potential coinfections with RSV,^34,43–46^ which could lead to underestimating RSV’s contribution if RSV increases hosts’ susceptibility to other pathogens. To address this, we evaluated the impact of excluding pathogens highly correlated with RSV (>0.7) on estimated age-specific RSV-attributable proportions. Furthermore, we assessed the impact of removing all respiratory pathogens except influenza and RSV on age-specific RSV-attributable proportions, as previous studies of RSV-attributable antibiotic prescriptions in the UK have only controlled for influenza.^2,3^

## Results

**Figure 1.**
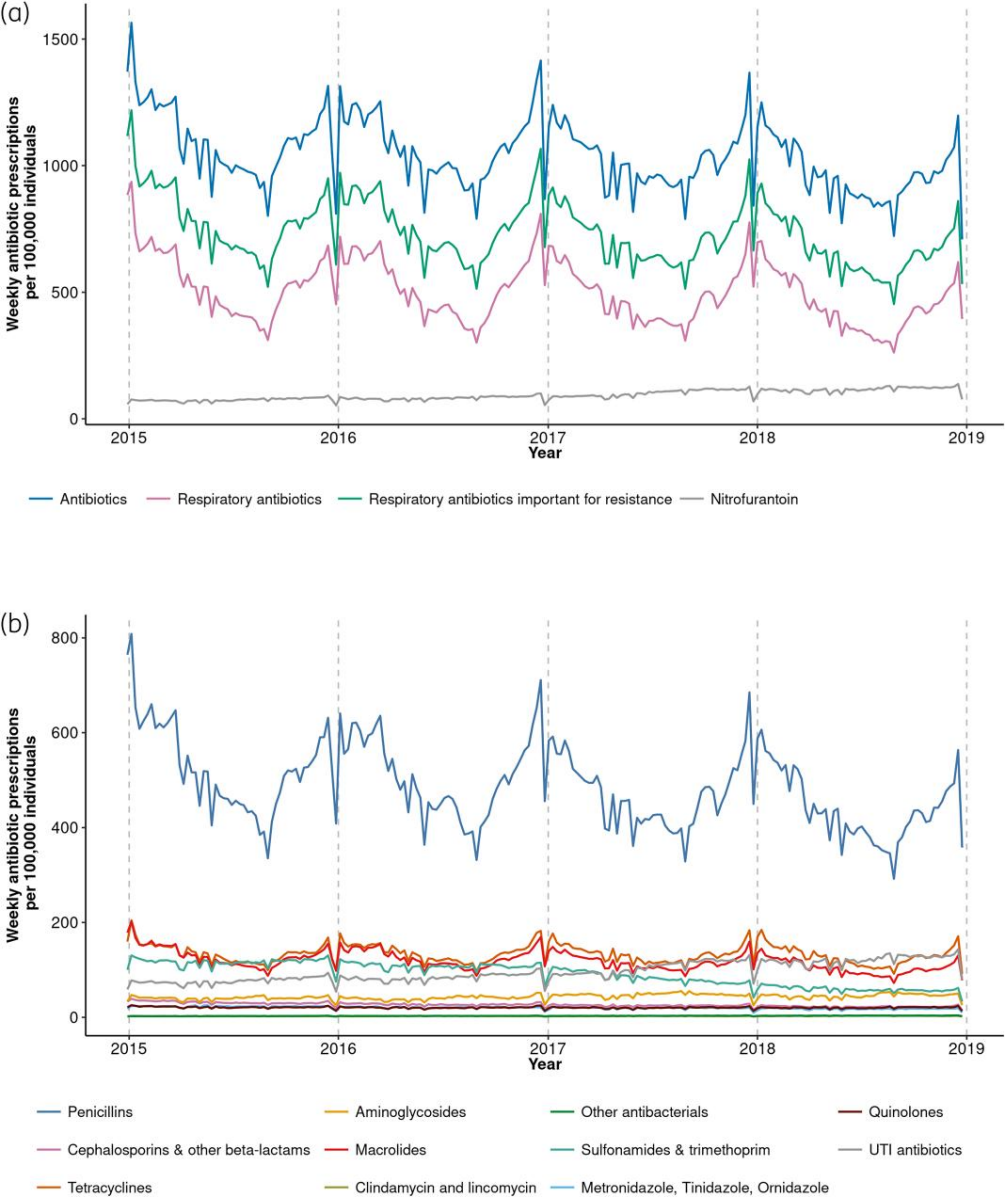
Weekly antibiotic prescriptions per 100 000 individuals in CPRD from calendar Week 1 2015 (29 December 2014) to calendar Week 52 2018 (30 December 2018). (a) antibiotic prescription outcomes; (b) classes of antibiotic prescriptions. Vertical dashed lines represent the start of a new calendar year.

Over the 4-year study period, 27 969 054 antibiotic prescriptions from 17 505 438 CPRD-registered patients were analysed, with 192 938 laboratory-confirmed respiratory infections (RSV = 36 180) and average weekly temperatures of median 10.3°C (IQR 6.6–14.6) (Tables S3–S4). Antibiotic and respiratory antibiotic prescription rates per year decreased from 584 and 287 per 1000 individuals in 2015 to 501 and 232 per 1000 individuals in 2018 (Table S3). Respiratory antibiotics comprised approximately half of all antibiotic prescriptions (Table 1), with penicillins most frequently prescribed for all ages (Figure 1 and Table S4). Respiratory antibiotic prescriptions were fewer than those important for resistance, reflecting the more conservative outcome definition for respiratory antibiotics (Section 1.1 in supplementary data). Infants 6–23 months had the highest rate of respiratory antibiotic prescriptions, while adults ≥75 years had the highest rate of antibiotic prescriptions (Table 1). Antibiotic and respiratory antibiotic prescriptions demonstrated winter seasonality, mainly driven by penicillins (Figure 1). All outcomes demonstrated matching dips in prescribing that correspond with practice bank holiday closures such as Christmas and Easter.

**Table 1.** Average annual antibiotic prescriptions and prescriptions per 100 000 in CPRD by age from 29 December 2014 to 30 December 2018

Age	Antibiotic prescriptions		Respiratory antibiotic prescriptions			Respiratory antibiotic prescriptions potentially important for resistance			Nitrofurantoin prescriptions (negative control)		
	Number	Rate per 100 000	Number	Rate per 100 000	%	Number	Rate per 100 000	%	Number	Rate per 100 000	%
0–5 months	11 712	30 462	7 250	18 849	62	10 202	26 528	87	29	75	<1
6–23 months	190 175	79 088	156 692	65,164	82	181 445	75 458	95	422	176	<1
2–4 years	283 890	59 847	216 771	45 696	76	258 962	54 591	91	1 006	212	<1
5–14 years	462 970	31 488	286 549	19 506	62	384 153	26,410	83	5 180	350	1
15–44 years	1 940 258	37 319	860 118	16 556	44	1 287 830	24 781	66	167 271	3 191	9
45–64 years	1 711 041	52 381	810 115	24 816	47	1 196 182	36 633	70	158 714	4 832	9
65–74 years	1 022 016	87 552	473 456	40 577	46	715 019	61 269	70	110 386	9 417	11
≥75 years	1 370 201	136,942	546 854	54 669	40	890 511	89 015	65	187 095	18 627	14
Total in CPRD	6 992 263		3 357 805		48	4 924 304		70	630 103		9

% = The age-specific proportion of outcome counts out of total antibiotic prescriptions. Respiratory antibiotic prescriptions included amoxicillin, phenoxymethylpenicillin, clarithromycin, erythromycin and doxycycline. Respiratory antibiotic prescriptions potentially important for resistance included amoxicillin, co-amoxiclav, phenoxymethylpenicillin, flucloxacillin, cefalexin, doxycycline, gentamicin, erythromycin, clarithromycin, azithromycin, levofloxacin, ciprofloxacin and co-trimoxazole (Table S1).

**Figure 2.**
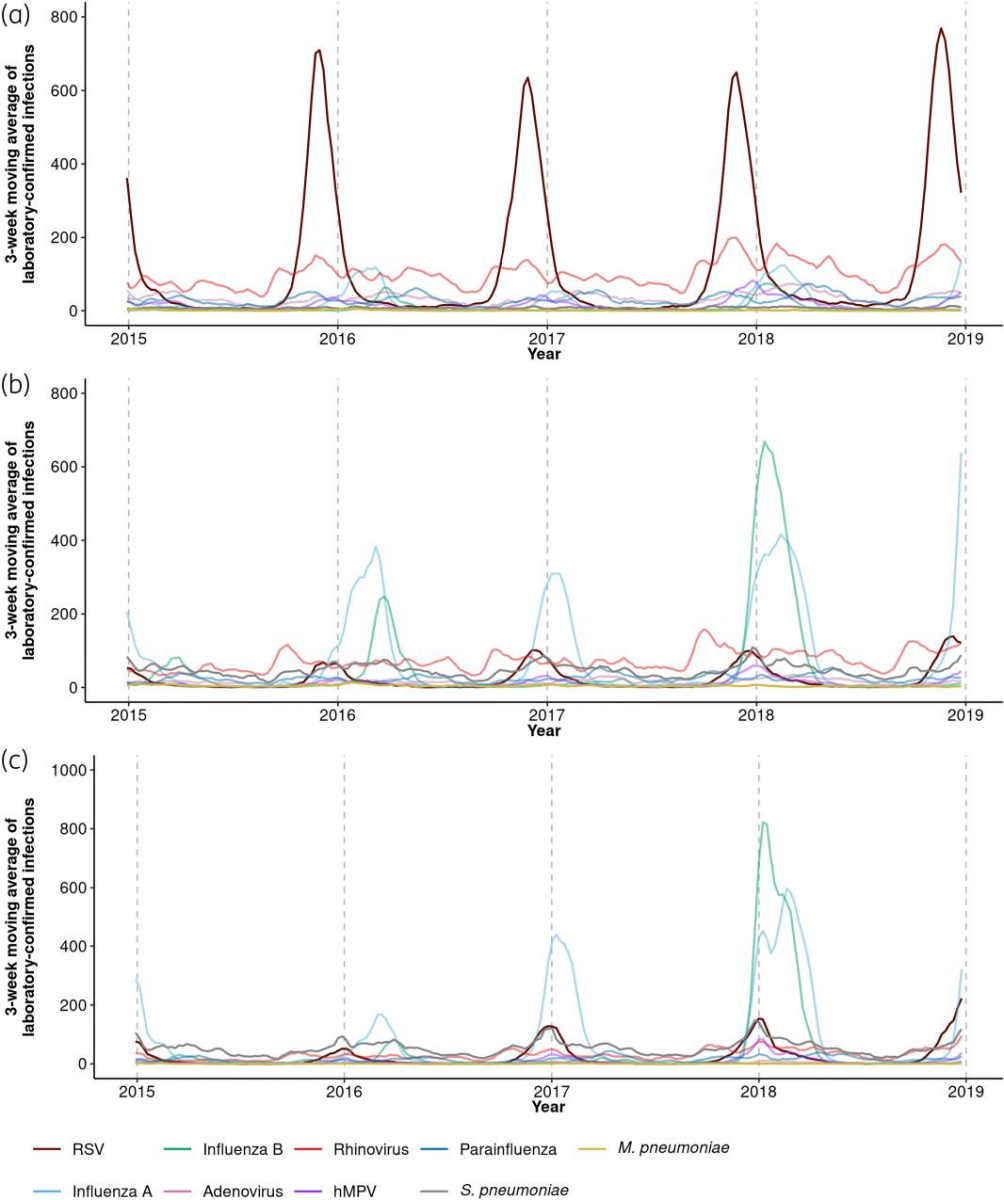
Three-week moving averages of laboratory-confirmed respiratory infections in England recorded in SGSS from calendar Week 1 2015 (29 December 2014) to calendar Week 52 2018 (30 December 2018) for individuals aged 0–4 years (a), 5–64 years (b) and ≥65 years (c). Vertical dashed lines represent the start of a new calendar year.

**Figure 3.**
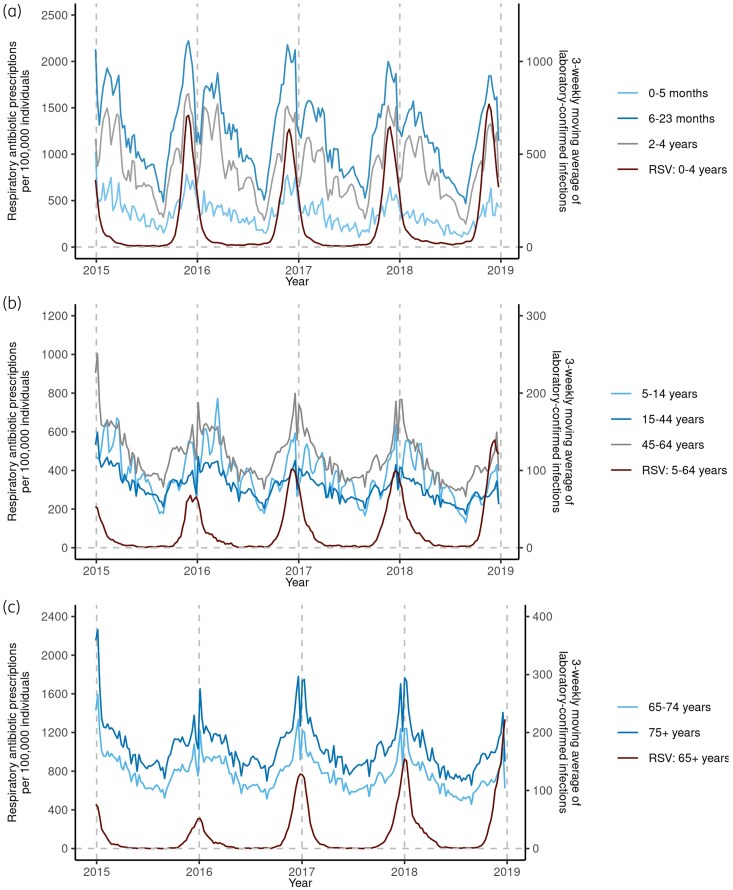
Rates of age-specific respiratory antibiotic prescriptions and corresponding laboratory-confirmed RSV infections in broader age groups from calendar Week 1 2015 (29 December 2014) to calendar Week 52 2018 (30 December 2018). Vertical dashed lines represent the start of a new calendar year.

Figures S1 and 2 demonstrate the seasonality of RSV and respiratory pathogens across three broad age groups: 0–4, 5–64 and ≥65 years. Around 74% of laboratory-confirmed RSV infections came from children <5 years, with sharp winter peaks observed for all ages. Peaks occurred later with increasing age, from late November for children <5 years to early January for adults ≥65 years (Figure S1). RSV dominated laboratory-confirmed respiratory infections from children <5 years during early winter, while influenza constituted most infections from individuals ≥5 years during late winter (Figure 2). Across most age groups used for antibiotic prescriptions, peaks of RSV infections in broader age groups aligned (Figure 3). RSV infections for 5–14 years peaked 1 week earlier than in 564 years (Figure S2).

Weekly respiratory infections of broad age groups demonstrated negative correlation with average temperatures in England (Figure S3). Most pathogens had low-to-moderate correlation with RSV across all age groups (Figure S3). High collinearity (>0.7) with RSV was observed for hMPV and *S. pneumoniae* in adults ≥65 years and for hMPV in individuals 5–64 years.

### RSV-attributable antibiotic prescribing

An estimated 2.1% of all antibiotic prescriptions and 4.3% of respiratory antibiotic prescriptions were attributable to RSV infections across all ages, amounting to an annual average of 639 908 GP prescriptions in England. Infants 6–23 months had the highest rates of RSV-attributable prescriptions, with an annual average of 6580 prescriptions per 100 000 individuals (95% CrI: 4522–8651) (Table 2). Adults ≥75 years had the highest annual volume of RSV-attributable prescriptions at 149 078 (95% CrI: 93 733–206 045). The secondary outcomes demonstrated a greater pool of antibiotic prescriptions, beyond those typically used for RTIs, associated with RSV infections in adults ≥45 years, with most attributable prescriptions across all ages likely important for resistance selection and development (Table S5).

**Table 2.** Average annual RSV-attributable GP respiratory antibiotic prescriptions in England, stratified by age and based on separate age-specific models of respiratory antibiotic prescriptions from 29 December 2014 to 30 December 2018

Average annual RSV-attributable GP respiratory antibiotic prescriptions in England				
Age	Prescriptions (95% CrI)	%	Attributable proportion % (95% CrI)	Rate per 100 000 (95% CrI)
0–5 m	6880^a^ (3211–10 167)	1.08	11 (5–16)	2100 (984–3100)
6–23 m	65 535^a^ (45 035–86 144)	10.24	10 (7–13)	6580 (4522–8651)
2–4 y	72 500 (29 222–119 821)	11.33	8 (3–13)	3497 (1405–5782)
5–14 y^b^	55 860 (−41 759 to 156 160)	8.73	4 (−3 to 12)	857 (−637 to 2397)
15–44 y	95 554 (6792–185 445)	14.93	3 (0–5)	447 (31–868)
45–64 y	86 608 (−16 608 to 191 877)	13.53	2 (0–6)	612 (−117 to 1357)
65–74 y	107 893 (60 407–158 507)	16.86	5 (3–7)	1972 (1104–2901)
≥75 y	149 078 (93 733–206 045)	23.30	6 (4–8)	3279 (2050–4532)

%, age-specific proportion; CrI, credible interval; m, months; y, years.

^a^Prescriptions are estimated assuming English mid-year populations are equally distributed by month of age as ONS population estimates are only provided by year of age.

^b^Average annual RSV-attributable prescriptions for the 5–14 age group were estimated from 2015 to 2017 and assumed to apply to 2018, as 2018 data were excluded for this group (see Section 2.2 in supplementary data).

Adults ≥65 years had a broader spectrum of antibiotic classes associated with RSV than younger age groups (Table 3). Among children <5 years, penicillins and macrolides had the highest proportion attributable to RSV, with 9% (95% CrI: 6–13) and 7% (95% CrI: 3–10), respectively, for infants 6–23 months. For adults ≥65 years, tetracyclines had the highest proportion attributable to RSV, with 6% (95% CrI: 3–8) for ≥75 years. Across all ages, penicillins accounted for the most RSV-attributable prescriptions, followed by macrolides and tetracyclines (Table S6).

**Table 3.** Age-specific RSV-attributable GP antibiotic prescriptions by class described by RSV-attributable proportion, based on separate age-specific models of antibiotic classes

Age-specific RSV-attributable proportion % of GP antibiotic prescriptions by class (95% CrI)								
Class	0–5 m	6–23 m	2–4 y	5–14 y	15–44 y	45–64 y	65–74 y	≥ 75 y
PEN	9 (4–14)	9 (6–13)	7 (2–11)	3 (−4 to 11)	—	3 (1–5)	3 (1–6)	4 (2–6)
CEPH+	—	3 (−2 to 7)	4 (0–8)	—	—	—	2 (0–5)	2 (0–4)
TET	—	—	—	—	—	3 (0–5)	5 (2–7)	6 (3–8)
AMINO	—	−4 (−10 to 3)	—	—	2 (−1 to 6)	2 (−1 to 5)	3 (1–6)	4 (1–6)
MAC	2 (−8 to 11)	7 (3–10)	7 (3–10)	3 (−3 to 10)	—	2 (0–4)	4 (2–7)	5 (2–7)
CLI+	—	—	—	—	—	—	—	—
OTHER	—	—	—	—	—	1 (−2 to 4)	4 (0–7)	—
SULPH+	1 (−5 to 8)	—	1 (−2 to 4)	—	—	—	2 (0–4)	2 (0–4)
MTZ+	—	—	—	—	1 (−2 to 3)	1 (−1 to 3)	2 (−1 to 4)	1 (−1 to 4)
QUIN	—	—	—	—	—	—	—	3 (0–5)
UTI	—	—	—	—	4 (0–7)	3 (0–5)	2 (0–5)	2 (0–5)

m, months; y, years; CrI, credible interval; PEN, penicillin; CEPH+, cephalosporins and other beta lactams; TET, tetracyclines; AMINO, aminoglycosides; MAC, macrolides; CLI+, clindamycin and lincomycin; OTHER, other antibacterials; SULPH+, sulphonamides and trimethoprim; MTZ+, metronidazole, tinidazole and ornidazole; QUIN, quinolones; UTI, urinary tract infection antibiotics; —, the model was not run because age-specific counts of antibiotic classes were <1000 during the study period or demonstrated no relationship with confirmed RSV infections.

### Negative control and sensitivity analysis

The negative control analysis with nitrofurantoin prescriptions, exclusively for UTIs, as the outcome demonstrated a potential association with RSV infections in individuals 15–44 and ≥75 years (Table S7 and Figure S13).

Removing *S. pneumoniae* from models for adults ≥65 years and hMPV from models for 5–14 and 45–64 years, which were highly correlated with RSV infections, increased the estimated RSV contribution by one percentage point for adults ≥45 years (Table S8). Removing all pathogens apart from influenza and RSV increased the estimated RSV contribution by 1–14 percentage points for most ages, with the largest increase in children <5 years (Table S8).

## Discussion

### Principal findings

Our analyses estimated that 2.1% of antibiotic prescriptions in English GPs were attributable to RSV infections. Prescribing to adults ≥75 years contributed to the greatest degree, despite infants between 6 and 23 months having the highest estimated rate of RSV-attributable prescribing. This was driven by the greater population size and high antibiotic prescribing rates of older adults (Table 1). The antibiotic classes frequently attributed to RSV infections were those recommended for RTIs, e.g. penicillins, macrolides and tetracyclines.^21,24–29^ However, our study suggested that a broader spectrum of antibiotic classes was associated with RSV infections in older adults, potentially due to the increased challenges of diagnosing infections in this age group.^47^

### Strengths and weaknesses

To our knowledge, this study provides the first estimate of RSV-attributable primary care antibiotic prescriptions by antibiotic class for individuals ≥5 years in the UK, using nationally representative GP records and laboratory-confirmed respiratory infections. We are reasonably confident that CPRD data, one of the largest available primary care datasets, covering 23% of individuals in England and broadly reflecting national demographics regarding age, sex and deprivation, is representative of the English population.^18^ We controlled for a wide range of respiratory pathogens that could drive RTI antibiotic prescribing and stratified counts by age to reflect age-specific differences in pathogen seasonality (Figure 2). This was important given the higher correlation between respiratory infections in older adults (Figure S3). A key strength of our approach lies in using GAMs, which provided robust variable selection and fitting. GAMs effectively penalized variables unrelated to antibiotic prescribing while considering other relationships in a single step, minimizing bias typically introduced by popular stepwise regression techniques.^36^ Additionally, GAMs allowed for more flexible adjustment of unmeasured seasonal confounding by fitting data-derived trends^36^ instead of assuming fixed cyclic patterns that could introduce bias.

We identified three previous studies estimating RSV-attributable antibiotic prescribing in the UK.^2–4^ Taylor *et al*.^2^ and Fleming *et al*.^3^ using CPRD data from 1995 to 2009 and only controlling for influenza reported higher proportions of RSV-attributable prescribing compared with our analysis. They estimated that 14.6% of respiratory antibiotic prescriptions in infants 6–23 months were attributable to RSV, compared with 10% in our study. In adults 64–75 and ≥75 years, they found 6% and 6.3% of respiratory antibiotic prescriptions attributable to RSV compared with our estimates of 5% and 6%. Our more conservative estimates likely reflect adjustments for additional respiratory pathogens in younger age groups, where controlling for pathogens beyond influenza was suggested to decrease RSV-attributable antibiotic prescriptions by up to 14 percentage points in children <5 years (Table S8). Additionally, GP antibiotic prescriptions significantly declined between study periods.^48^

Fitzpatrick *et al.*^4^ analysed data for children <5 years in Scotland from 2009 to 2017, associating laboratory tests of multiple respiratory pathogens with all community antibiotic prescriptions, including those from additional providers like dentists. They reported similar proportions of RSV-attributable antibiotic prescriptions for children <5 years (6.92%) compared with our secondary analysis of GP-only prescriptions (6–8%, Table S5). Scaling our results to average community antibiotic prescribing for the study period to match the denominator used by Fitzpatrick *et al.*^4^ gives a comparable 5.2–6.9% for children <5 years.^48^ They also estimated comparable RSV-attributable proportions for penicillin (amoxicillin) and macrolide prescriptions in this age group (8.1% and 7.7% versus 7%–9% and 2%–7% in our study). Our study is the first to estimate age-specific RSV-attributable proportions for antibiotic classes beyond those typically prescribed for RTIs and across all ages.

Our study’s annual rates of antibiotic and respiratory antibiotic prescriptions in GPs were slightly lower than previously reported for 2015–18. The English Surveillance Programme for Antimicrobial Utilisation and Resistance report estimated 602 to 532 per 1000 individuals in English GPs between 2015 and 2018, compared with our rates of 584 to 501 per 1000 individuals.^48^ Similarly, national analyses of respiratory antibiotic prescriptions in the community, irrespective of prior diagnosis, estimated quarterly rates of 65–100 per 1000 individuals,^15^ while our average quarterly rates were ∼58–72 per 1000 individuals. These lower rates likely reflect our more conservative outcome definitions, which excluded topical antibiotics (except for ear infections) from the overall count and certain respiratory antibiotics, such as co-amoxiclav, commonly used for other conditions (Section 1.1 in supplementary data).^21^ Co-amoxiclav had quarterly community dispensing rates of ∼6–8.25 per 1000 individuals during this period.^49^

Common limitations of studies utilizing laboratory data of respiratory pathogens include underreporting and most tests likely being from hospital cases, which may not represent the study population. Biases may arise from changes in testing practices, healthcare-seeking behaviours or discrepancies between our age groups for surveillance and prescription data. For example, the 5–14-year model struggled with 2018 data (Figure S12), likely due to a significant increase in influenza samples in 5–64 years, more representative of adults (Figure 2).^34^

The study could not control for possible RSV coinfections, potentially underestimating RSV’s contribution. Sensitivity analysis suggested that including hMPV or *S. pneumoniae*, both highly correlated with RSV in adults ≥45 years, may have underestimated RSV-attributable antibiotic prescriptions by 1 percentage point in this age group. Coinfections with *S. pneumoniae* and hMPV are linked to increased disease severity,^44,50^ and RSV may enhance the susceptibility and virulence of *S. pneumoniae*;^51^ thus, results for older adults may be conservative. However, coinfection frequency in the general population remains poorly understood, as most evidence is based on co-detections from symptomatic samples, which are prone to bias.^52,53^ Country-specific, community-based studies that collect respiratory pathogen samples regardless of clinical status,^52^ accounting for factors such as viral load (to indicate infection),^53^ age and comorbidities, are needed to understand infection and coinfection incidence better.

Finally, unmeasured confounding may lead to overestimated model predictions. The negative control demonstrated an association between RSV infections and nitrofurantoin prescriptions for individuals 15–44 and ≥75 years. In 15–44 years, this association may indicate unaccounted-for confounding due to overlapping seasonality of RTIs and UTIs at the start of university periods,^54^ suggesting a potential overestimation of the RSV contribution in this group. However, in adults ≥75 years, winter peaks in nitrofurantoin prescriptions do not likely match UTI activity.^54^ Instead, these peaks may be due to older adults with RTIs being treated for UTIs, reflecting non-specific symptoms and diagnostic challenges in identifying a source of infection,^55,56^ highlighting the difficulties of antibiotic stewardship in this group.^47,55^

### Implications and conclusions

Our study suggests that interventions like vaccines or mABs to reduce the burden of RSV infections in England could complement national efforts to reduce antibiotic use.^1^ The largest potential reductions are in older adults, an age group for whom antibiotic stewardship is challenging.^47,55^

The full impact of RSV prescribing reductions on AMR remains unclear. Most RSV-attributable prescriptions were ‘Access’ antibiotics, such as amoxicillin and doxycycline, recommended by the WHO as first- and second-line treatments for common infections and considered to have lower resistance potential.^57^ However, extensively used antibiotics like amoxicillin may affect commensal pathogens systematically,^11^ with evidence of potentially promoting the co-selection of resistant UTIs in the community.^12,58^ This study was unable to explore secondary care antibiotic prescriptions, which, though smaller in volume, typically involve broader spectrum antibiotics with higher resistance potential^9^ and are used in patients at greater risk of severe resistant infections.

Despite these limitations, our findings provide a prerequisite for exploring the onward impacts of reduced RSV-related prescribing on AMR and can inform future modelling.

## Supplementary Material

dkaf043_Supplementary_Data

## Data Availability

This study is based in part on data from the CPRD obtained under licence from the UK Medicines and Healthcare products Regulatory Agency. The data were provided by patients and collected by the NHS as part of their care and support. Data from the SGSS were provided by the UKHSA under licence. Both datasets are not publicly available. However, an application for CPRD data can be made to the Independent Scientific Advisory Committee and SGSS data can be requested via the office of data release at UKHSA.

## Supplementary data

Figures S1–S13, Equation S1 and Tables S1–S8 are available as Supplementary data at *JAC* Online.

## References

[1] Global and Public Health Group, Emergency Preparedness and Health Protection, Policy Directorate . Confronting Antimicrobial Resistance 2024 to 2029. 2024. https://www.gov.uk/government/publications/uk-5-year-action-plan-for-antimicrobial-resistance-2024-to-2029/confronting-antimicrobial-resistance-2024-to-2029

[2] Taylor S, Taylor RJ, Lustig RL et al Modelling estimates of the burden of respiratory syncytial virus infection in children in the UK. BMJ Open 2016; 6: e009337. 10.1136/bmjopen-2015-009337 PMC4893852 27256085

[3] Fleming DM, Taylor RJ, Lustig RL et al Modelling estimates of the burden of respiratory syncytial virus infection in adults and the elderly in the United Kingdom. BMC Infect Dis 2015; 15: 443. 10.1186/s12879-015-1218-z 26497750 PMC4618996

[4] Fitzpatrick T, Malcolm W, McMenamin J et al Community-based antibiotic prescribing attributable to respiratory syncytial virus and other common respiratory viruses in young children: a population-based time series study of Scottish children. Clin Infect Dis 2021; 72: 2144–53. 10.1093/cid/ciaa403 32270199

[5] Walsh EE . Respiratory syncytial virus infection: an illness for all ages. Clin Chest Med 2017; 38: 29–36. 10.1016/j.ccm.2016.11.010 28159159 PMC5844562

[6] UK Health Security Agency . Chapter 27a: Respiratory syncytial virus. The Green Book. https://assets.publishing.service.gov.uk/media/669a5e37ab418ab05559290d/Green-book-chapter-27a-RSV-18_7_24.pdf

[7] Vekemans J, Hasso-Agopsowicz M, Kang G et al Leveraging vaccines to reduce antibiotic use and prevent antimicrobial resistance: a World Health Organization action framework. Clin Infect Dis 2021; 73: e1011–7. 10.1093/cid/ciab062 33493317 PMC8366823

[8] Lewnard JA, Fries LF, Cho I et al Prevention of antimicrobial prescribing among infants following maternal vaccination against respiratory syncytial virus. Proc Natl Acad Sci U S A 2022; 119: e2112410119. 10.1073/pnas.2112410119 35286196 PMC8944586

[9] UK Health Security Agency . English surveillance programme for antimicrobial utilisation and resistance (ESPAUR): Report 2022 to 2023. https://webarchive.nationalarchives.gov.uk/ukgwa/20240201222414mp_/https://assets.publishing.service.gov.uk/media/6555026e544aea000dfb2e19/ESPAUR-report-2022-to-2023.pdf

[10] Shi T, Denouel A, Tietjen AK et al Global disease burden estimates of respiratory syncytial virus–associated acute respiratory infection in older adults in 2015: a systematic review and meta-analysis. J Infect Dis 2020; 222: S577–83. 10.1093/infdis/jiz059 30880339

[11] Tedijanto C, Olesen SW, Grad YH et al Estimating the proportion of bystander selection for antibiotic resistance among potentially pathogenic bacterial flora. Proc Natl Acad Sci U S A 2018; 115: E11988–95. 10.1073/pnas.1810840115 30559213 PMC6304942

[12] Pouwels KB, Muller-Pebody B, Smieszek T et al Selection and co-selection of antibiotic resistances among Escherichia coli by antibiotic use in primary care: an ecological analysis. PLoS One 2019; 14: e0218134. 10.1371/journal.pone.0218134 31181106 PMC6557515

[13] UK Health Security Agency . Surveillance of influenza and other seasonal respiratory viruses in the UK, winter 2022 to 2023. https://www.gov.uk/government/statistics/annual-flu-reports/surveillance-of-influenza-and-other-seasonal-respiratory-viruses-in-the-uk-winter-2022-to-2023

[14] Bardsley M, Morbey RA, Hughes HE et al Epidemiology of respiratory syncytial virus in children younger than 5 years in England during the COVID-19 pandemic, measured by laboratory, clinical, and syndromic surveillance: a retrospective observational study. Lancet Infect Dis 2023; 23: 56–66. 10.1016/S1473-3099(22)00525-4 36063828 PMC9762748

[15] Andrews A, Bou-Antoun S, Guy R et al Respiratory antibacterial prescribing in primary care and the COVID-19 pandemic in England, winter season 2020–21. J Antimicrob Chemother 2022; 77: 799–802. 10.1093/jac/dkab443 34897486 PMC9383059

[16] [Dataset] Clinical Practice Research Datalink (2021). CPRD Aurum June 2021 (Version 2021.06.001). Clinical Practice Research Datalink. 10.48329/pyc2-we97

[17] [Dataset] Office for National Statistics (ONS) . 2021. Estimates of the population for the UK, England and Wales, Scotland and Northern Ireland. https://www.ons.gov.uk/peoplepopulationandcommunity/populationandmigration/populationestimates/datasets/populationestimatesforukenglandandwalesscotlandandnorthernireland

[18] Wolf A, Dedman D, Campbell J et al Data resource profile: Clinical Practice Research Datalink (CPRD) Aurum. Int J Epidemiol 2019; 48: 1740–G. 10.1093/ije/dyz034 30859197 PMC6929522

[19] Clinical Practice Research Datalink . CPRD linked data. https://www.cprd.com/cprd-linked-data

[20] Clinical Practice Research Datalink . Data quality. https://www.cprd.com/data-quality

[21] Dolk FCK, Pouwels KB, Smith DRM et al Antibiotics in primary care in England: which antibiotics are prescribed and for which conditions? J Antimicrob Chemother 2018; 73: ii2–10. 10.1093/jac/dkx504 29490062 PMC5890730

[22] WHO Collaborating Centre for Drug Statistics Methodology . ATC/DDD Index. https://www.whocc.no/atc_ddd_index/? code=J01&showdescription=no

[23] OpenPrescribing.net, Bennett Institute for Applied Data Science, University of Oxford . 5.1: Antibacterial Drugs. 2025. https://openprescribing.net/bnf/0501/

[24] National Institute for Health and Care Excellence (NICE) . Otitis media (acute): antimicrobial prescribing. NICE guideline [NG91]. 2018. https://www.nice.org.uk/guidance/ng91

[25] National Institute for Health and Care Excellence (NICE ). Cough (acute): antimicrobial prescribing. NICE guideline [NG120]. 2019. https://www.nice.org.uk/guidance/ng120

[26] National Institute for Health and Care Excellence (NICE) . Sinusitis (acute): antimicrobial prescribing. NICE guideline [NG79]. 2017. https://www.nice.org.uk/guidance/ng79

[27] National Institute for Health and Care Excellence (NICE) . Sore throat (acute): antimicrobial prescribing. NICE guideline [NG84]. 2018. https://www.nice.org.uk/guidance/ng84

[28] National Institute for Health and Care Excellence (NICE) . Pneumonia (community-acquired): antimicrobial prescribing. NICE guideline [NG138]. 2019. https://www.nice.org.uk/guidance/ng138/chapter/recommendations#choice-of-antibiotic

[29] National Institute for Health and Care Excellence (NICE) . Bronchiolitis in children: diagnosis and management. NICE guideline [NG9]. 2015. https://www.nice.org.uk/guidance/ng9

[30] National Institute for Health and Care Excellence (NICE) . Urinary tract infection (lower): antimicrobial prescribing. NICE guideline [NG109]. 2018. https://www.nice.org.uk/guidance/ng109

[31] Holmes AH, Moore LSP, Sundsfjord A et al Understanding the mechanisms and drivers of antimicrobial resistance. Lancet 2016; 387: 176–87. 10.1016/S0140-6736(15)00473-0 26603922

[32] UK Health Security Agency . English surveillance programme for antimicrobial utilisation and resistance (ESPAUR): Report 2020 to 2021. https://webarchive.nationalarchives.gov.uk/ukgwa/20221020175458mp_/https://assets.publishing.service.gov.uk/government/uploads/system/uploads/attachment_data/file/1069632/espaur-report-2020-to-2021-16-Nov-FINAL-v2.pdf

[33] UK Health Security Agency . Laboratory reporting to UKHSA: A guide for diagnostic laboratories. https://assets.publishing.service.gov.uk/media/66e2e0ba0d913026165c3d77/UKHSA_Laboratory_reporting_guidelines_May_2023.pdf

[34] Tanner H, Boxall E, Osman H. Respiratory viral infections during the 2009–2010 winter season in Central England, UK: incidence and patterns of multiple virus co-infections. Eur J Clin Microbiol Infect Dis 2012; 31: 3001–6. 10.1007/s10096-012-1653-3 22678349 PMC7088042

[35] Parker DE, Legg TP, Folland CK. A new daily Central England temperature series, 1772–1991. Intern J Climatol 1992; 12: 317–42. 10.1002/joc.3370120402

[36] Marra G, Wood SN. Practical variable selection for generalized additive models. Comput Stat Data Anal 2011; 55: 2372–87. 10.1016/j.csda.2011.02.004

[37] Desphande SA, Northern V. The clinical and health economic burden of respiratory syncytical virus disease among children under 2 years of age in a defined geographical area. Arch Dis Child 2003; 88: 1065–9. 10.1136/adc.88.12.1065 14670770 PMC1719378

[38] Elliott SP, Ray CG. Viral infections of the lower respiratory tract. Pediatr Respir Med 2009; 18: 481–9. 10.1016/B978-032304048-8.50037-2

[39] NHS . Respiratory tract infections (RTIs). https://www.nhs.uk/conditions/respiratory-tract-infection/

[40] Wood SN . mgcv: Mixed GAM Computation Vehicle with Automatic Smoothness Estimation (version 1.8-4.0) [R package]. 2022. https://cran.r-project.org/web/packages/mgcv/index.html

[41] Fasiolo M, Nedellec R., Goude Y, et al mgcViz: Visualisations for Generalized Additive Models. (version 0.1.11) [R package]. 2018. https://CRAN.R-project.org/package=mgcViz

[42] Wood SN . Generalized Additive Models: An Introduction with R. CRC Press LLC, 2006.

[43] Pacheco GA, Gálvez NMS, Soto JA et al Bacterial and viral coinfections with the human respiratory syncytial virus. Microorganisms 2021; 9: 1293. 10.3390/microorganisms9061293 34199284 PMC8231868

[44] Diaz-Diaz A, Bunsow E, Garcia-Maurino C et al Nasopharyngeal codetection of Haemophilus influenzae and Streptococcus pneumoniae shapes respiratory syncytial virus disease outcomes in children. J Infect Dis 2022; 225: 912–23. 10.1093/infdis/jiab481 34543409 PMC8889286

[45] Liu Y, Ling L, Wong SH et al Outcomes of respiratory viral-bacterial co-infection in adult hospitalized patients. EClinicalMedicine 2021; 37: 100955. 10.1016/j.eclinm.2021.100955 34386745 PMC8343259

[46] Choo S, Lee YY, Lee E. Clinical significance of respiratory virus coinfection in children with Mycoplasma pneumoniae pneumonia. BMC Pulm Med 2022; 22: 212. 10.1186/s12890-022-02005-y 35637540 PMC9150047

[47] Hayward GN, Moore A, Mckelvie S et al Antibiotic prescribing for the older adult: beliefs and practices in primary care. J Antimicrob Chemother 2019; 74: 791–7. 10.1093/jac/dky504 30566597

[48] Public Health England . English surveillance programme for antimicrobial utilisation and resistance (ESPAUR): Report 2019 to 2020. https://webarchive.nationalarchives.gov.uk/ukgwa/20211022024510mp_/https://assets.publishing.service.gov.uk/government/uploads/system/uploads/attachment_data/file/936199/ESPAUR_Report_2019-20.pdf

[49] OpenPrescribing.net, Bennett Institute for Applied Data Science, University of Oxford . Long Term Trends. 2025. https://openprescribing.net/long_term_trends/

[50] Li Y, Pillai P, Miyake F et al The role of viral co-infections in the severity of acute respiratory infections among children infected with respiratory syncytial virus (RSV): a systematic review and meta-analysis. J Glob Health 2020; 10: 010426. 10.7189/jogh.10.010426 32566164 PMC7295447

[51] Besteman SB, Bogaert D, Bont L et al Interactions between respiratory syncytial virus and *Streptococcus pneumoniae* in the pathogenesis of childhood respiratory infections: a systematic review. Lancet Respir Med 2024; 12: 915–32. 10.1016/S2213-2600(24)00148-6 38991585

[52] Chin T, Foxman EF, Watkins TA et al Considerations for viral co-infection studies in human populations. mBio 2024; 15: e0065824. 10.1128/mbio.00658-24 38847531 PMC11253623

[53] Burstein R, Althouse BM, Adler A, et al Interactions among 17 respiratory pathogens: a cross-sectional study using clinical and community surveillance data. medRxiv 22270474. 10.1101/2022.02.04.22270474, 6 February 2022, preprint: not peer reviewed.

[54] Rosello A, Pouwels KB, Domenech De Cellès M, et al Seasonality of urinary tract infections in the United Kingdom in different age groups: longitudinal analysis of The Health Improvement Network (THIN). Epidemiol Infect 2018; 146: 37–45. 10.1017/S095026881700259X 29168442 PMC9134528

[55] Beckett CL, Harbarth S, Huttner B. Special considerations of antibiotic prescription in the geriatric population. Clin Microbiol Infect 2015; 21: 3–9. 10.1016/j.cmi.2014.08.018 25636920

[56] Anand Misra V, Oliver Hamilton D, Subudhi C. Difficulties in diagnosis and treatment of urinary tract infections in an elderly population. Access Microbiol 2019; 1: 457. 10.1099/acmi.ac2019.po0272

[57] WHO Essential Medicines Team . The WHO AWaRe (Access, Watch, Reserve) Antibiotic Book. World Health Organization, 2022. https://www.who.int/publications/i/item/9789240062382

[58] Pouwels KB, Freeman R, Muller-Pebody B et al Association between use of different antibiotics and trimethoprim resistance: going beyond the obvious crude association. J Antimicrob Chemother 2018; 73: 1700–7. 10.1093/jac/dky031 29394363

