# Supplementary material for: General practice antibiotic prescriptions attributable to respiratory syncytial virus by age and antibiotic class: an ecological analysis of the English population
Source: J Antimicrob Chemother. 2025 Feb 19;80(4):1116–26. doi: 10.1093/jac/dkaf043 (PMC11962385; doi:10.1093/jac/dkaf043)
Supplement: dkaf043_Supplementary_Data [file dkaf043_supplementary_data.docx]

**Supplementary data to “General practice antibiotic prescriptions attributable to Respiratory Syncytial Virus by age and antibiotic class: An ecological analysis of the English population”**

1. **Methods:**
2. **Definition of outcomes**
3. **Study population**
4. **Initial model equation**
5. **Results:**
6. **Descriptive results**
7. **Model fitting**
8. **Secondary outcomes and negative control**
9. **Methods**

**1.1 Definition of outcomes**

Classes of antibiotics are defined by the British National Formulary (BNF) Chapter 5.1 subsections^1^ including;

- Penicillins
- Cephalosporins and other beta-lactams
- Tetracyclines
- Aminoglycosides
- Macrolides
- Clindamycin and Lincomycin
- Other antibacterials
- Sulfonamides and Trimethoprim
- Metronidazole, Tinidazole and Ornidazole
- Quinolones
- UTI antibiotics

Respiratory antibiotic prescriptions are specified as antibiotics that are most frequently used to treat respiratory tract infections (RTIs) in primary care with only a small overlap of use for other non-respiratory conditions,^2–8^ namely: amoxicillin, phenoxymethylpenicillin, clarithromycin, erythromycin and doxycycline. Respiratory antibiotics potentially important for resistance (Table S1) were defined as antibiotics that are recommended in National Institute for Health and Care Excellence (NICE) treatment guidance for RTIs and ear infections,^2–8^ and have either:

1. Evidence of clinically relevant resistance in bacteria of high carriage prevalence in the UK, according to recent ESPAUR reports.
2. Evidence of clinically relevant resistance as described in (i), where most of the bacteria’s exposure to the antibiotic is potentially from bystander exposure.^9^
3. Evidence of driving clinically relevant resistance (i) to other antibiotics via co-selection.
4. Evidence of a high volume of use in UK primary care, therefore a potential candidate of co-selection for organisms of high carriage prevalence in the UK.

Nitrofurantoin prescriptions typically only used for treating urinary tract infections (UTIs)^2,10^ were used as a negative control to explore unaccounted-for residual confounding. A practising pharmacist and clinical microbiologist reviewed finalised lists of respiratory antibiotics and respiratory antibiotics potentially important for resistance. A practicing general practitioner reviewed finalised lists of antibiotic outcomes.

**Table S1:** Finalised list of respiratory antibiotics potentially important for resistance in the UK

| **Respiratory antibiotics** | **Clinically relevant resistance in the UK** | | | | **Co-selection of resistance** | | **High volume** | **Reference** |
| --- | --- | --- | --- | --- | --- | --- | --- | --- |
|  | ***E. coli*** | ***K. pneumoniae*** | ***S. pneumoniae*** | ***S. aureus*** | ***E. coli*** | ***S. aureus*** |  |  |
| **Penicillins** | | | | | | | |  |
| Co-amoxiclav | **x** | **x** | **x** |  |  |  |  | ^11,12^ |
| Amoxicillin |  |  | **x** |  | **x** |  |  | ^2,11–14^ |
| Phenoxymethylpenicillin |  |  |  |  |  |  | **x** | ^2,11,12^ |
| Flucloxacillin |  |  |  |  |  |  | **x** | ^2^ |
| **Cephalosporins and other beta-lactams** | | | | | | | | |
| Cefalexin | **x** | **x** |  |  |  |  |  | ^11,12^ |
| **Tetracyclines** | | | | | | | | |
| Doxycycline |  |  | **x** |  |  |  |  | ^2,11,12^ |
| **Aminoglycosides** | | | | | | | | |
| Gentamicin | **x** | **x** |  |  |  |  |  | ^11,12^ |
| **Macrolides** | | | | | | | | |
| Erythromycin |  |  | **x** |  |  |  |  | ^11,12^ |
| Clarithromycin |  |  | **x** | **x** |  |  | **x** | ^2,11,12^ |
| Azithromycin |  |  | **x** |  |  |  |  | ^11,12^ |
| **Fluoroquinolones** | | | | | | | | |
| Levofloxacin |  |  |  |  |  | **x** |  | ^15^ |
| Ciprofloxacin | **x** | **x** |  |  |  | **x** |  | ^11,12,15^ |
| **Sulfonamides and Trimethoprim** | | | | | | | | |
| Co-trimoxazole |  |  |  |  | **x** |  |  | ^13,16^ |

**1.2 Study population**

**Table S2:** Mid-year estimates of the CPRD study population compared to Office of National Statistics (ONS) mid-year population estimates for England ^17^ for the study period from 2015-2018.

| **Year** | **2015** | **2016** | **2017** | **2018** |
| --- | --- | --- | --- | --- |
| **Mid-year population in CPRD study population** | | | | |
| 0-5m | 38,142 | 39,432 | 38,611 | 37,640 |
| 6-23 m | 238,809 | 240,573 | 242,893 | 239,744 |
| 2-4 y | 476,680 | 474,485 | 472,586 | 473,330 |
| 5-14 y | 1,399,998 | 1,453,645 | 1,503,173 | 1,537,273 |
| 15-44y | 5,019,852 | 5,133,276 | 5,274,704 | 5,410,860 |
| 45-64y | 3,161,115 | 3,239,387 | 3,313,310 | 3,368,794 |
| 65-74y | 1,129,375 | 1,160,644 | 1,183,455 | 1,199,898 |
| ≥75 y | 972,435 | 987,963 | 1,011,551 | 1,033,952 |
| Total | 12,436,406 | 12,729,405 | 13,040,283 | 13,301,491 |
| **ONS mid-year population England** | | | | |
| Total | 54,786,327 | 55,268,067 | 55,619,430 | 55,977, 178 |
| Proportion (%) | 22.70 | 23.03 | 23.45 | 23.76 |

Mid-year population estimates for the study population represent the number of patient IDs on 1^st^ July for each year of the study period. Proportion = proportion of the mid-year English population represented by the mid-year CPRD study population. Y= years, m = months.

**1.3 Initial model equation**

**Equation S1**: Initial model equation

$$E\left( y_{t}^{a} \right)= \beta_{0}^{a}P_{t}^{a}+\beta_{1}^{a}RSVP_{t}^{a}+\beta_{2}^{a}InfluenzaP_{t}^{a}+\beta_{3}^{a}AdenovirusP_{t}^{a}+ \beta_{4}^{a}hMPVP_{t}^{a}+ \beta_{5}^{a}ParainfluenzaP_{t}^{a}+\beta_{6}^{a}RhinovirusP_{t}^{a}+\beta_{7}^{a}S.pneumoniaeP_{t}^{a}+\beta_{8}^{a}M.pneumoniaeP_{t}^{a}+\beta_{9}^{a}temperatureP_{t}^{a}$$

The basic model is represented above where ($E\left( y_{t}^{a} \right)$) represents the count of expected outcomes (e.g., respiratory antibiotic prescriptions) for each age group (a) in the week (t). The right side of the equation contains the intercept ($\beta_{0}^{a})$, weekly counts of respiratory pathogens and average temperatures, each multiplied by the mid-year population for age group (a), in the week (t) ($P_{t}^{a}$).

1. **Results**

**2.1 Descriptive results**

**Table S3:** Annual counts and rates of antibiotic and respiratory antibiotic prescriptions in CPRD and counts of laboratory-confirmed infections from SGSS over the study period from 2015-2018.

| **Year** | **2015** | **2016** | **2017** | **2018** | **Total** |
| --- | --- | --- | --- | --- | --- |
| Antibiotic prescriptions | 7,264,082 | 7,103,669 | 6,935,912 | 6,665,391 | 27,969,054 |
| Antibiotic prescriptions per 1,000 individuals | 584 | 558 | 532 | 501 | - |
| Respiratory antibiotic prescriptions | 3,566,383 | 3,488,283 | 3,292,600 | 3,083,950 | 13,431,216 |
| Respiratory antibiotic prescriptions per 1,000 individuals | 287 | 274 | 252 | 232 | - |
| Laboratory-confirmed respiratory infections | 33,602 | 42,784 | 44,755 | 71,797 | 192,938 |
| RSV | 8,123 | 8,200 | 8,449 | 11,408 | 36,180 |
| Influenza A | 3,464 | 8,538 | 7,844 | 16,347 | 36,193 |
| Influenza B | 1,410 | 3,120 | 2,362 | 14,194 | 21,086 |
| Adenovirus | 2,289 | 2,413 | 2,907 | 3,398 | 11,007 |
| Rhinovirus | 8,250 | 9,983 | 11,100 | 13,220 | 42,553 |
| hMPV | 1,244 | 1,453 | 1,933 | 2,696 | 7,326 |
| Parainfluenza | 3,212 | 2,989 | 4,202 | 4,366 | 14,769 |
| *S. pneumoniae* | 5,229 | 5,677 | 5,665 | 5,949 | 22,520 |
| *M. pneumoniae* | 381 | 411 | 293 | 219 | 1,304 |

**Table S4:** Proportion of total prescriptions in CPRD for age groups by class and WHO AWaRe category^18^ for study period 2015-2018.

| Age | 0-5m N=46,850 | 6-23m N=760,701 | 2-4y N=1,135,558 | 5-14y N=1,851,882 | 15-44y N=7,761,032 | 45-64y N=6,844,164 | 65-74y N=4,088,064 | ≥75y N=5,480,803 | Total N=27,969,054 |
| --- | --- | --- | --- | --- | --- | --- | --- | --- | --- |
| **Class %** | | | | | | | | | |
| PEN | 77.6 | 81.5 | 75.9 | 67.0 | 46.2 | 43.0 | 40.1 | 38.7 | 46.7 |
| CEPH+ | 2.4 | 1.3 | 1.5 | 1.6 | 1.7 | 1.9 | 3.0 | 4.9 | 2.5 |
| TET | <0.1 | <0.1 | <0.1 | 3.8 | 15.6 | 15.6 | 15.1 | 11.0 | 12.8 |
| AMINO | 1.5 | 0.9 | 2.2 | 5.4 | 4.5 | 5.3 | 4.3 | 2.7 | 4.2 |
| MAC | 7.9 | 12.4 | 13.5 | 13.0 | 10.6 | 11.8 | 12.2 | 10.2 | 11.4 |
| CLI+ | <0.1 | <0.1 | <0.1 | <0.1 | 0.2 | 0.3 | 0.3 | 0.4 | 0.2 |
| OTHER | 0.1 | 0.1 | 0.1 | 0.2 | 0.2 | 0.4 | 0.5 | 0.3 | 0.3 |
| SULF+ | 9.8 | 3.3 | 6.0 | 7.1 | 7.0 | 7.7 | 9.3 | 14.0 | 8.7 |
| MTZ+ | 0.3 | 0.1 | 0.2 | 0.3 | 3.6 | 2.1 | 1.4 | 1.0 | 1.9 |
| QUIN | 0.1 | 0.2 | 0.2 | 0.4 | 1.7 | 2.4 | 2.6 | 2.5 | 2.0 |
| UTI | 0.3 | 0.2 | 0.4 | 1.1 | 8.7 | 9.5 | 11.2 | 14.3 | 9.3 |
| **AWaRe Category %** | | | | | | | | | |
| Access | 91.7 | 87.0 | 84.3 | 78.6 | 75.3 | 77.4 | 79.6 | 84.8 | 79.3 |
| Watch | 8.3 | 13.0 | 15.6 | 21.2 | 24.5 | 22.3 | 19.8 | 14.6 | 20.4 |
| Reserve | <0.1 | <0.1 | 0.1 | 0.2 | 0.1 | 0.1 | 0.2 | 0.1 | 0.1 |
| NA | <0.1 | <0.1 | <0.1 | <0.1 | 0.1 | 0.2 | 0.4 | 0.5 | 0.2 |

N = number of antibiotic prescriptions in CPRD over the study period. m = months, y = years, NA = no category, PEN = Penicillin's, CEPH+ = Cephalosporins & other beta lactams, TET = Tetracyclines, AMINO = Aminoglycosides, MAC = Macrolides, CLI+ = Clindamycin & Lincomycin, OTHER = Other antibacterials, SULF+ = Sulfonamides & Trimethoprim, MTZ+ = Metronidazole, Tinidazole & Ornidazole, QUIN = Quinolones, UTI = Urinary tract infection antibiotics.

**Figure S1:** Three-week moving averages of laboratory-confirmed RSV infections in England recorded in SGSS from calendar week 1 2015 (29 December 2014) to calendar week 52 2018 (30 December 2018) stratified by age.

**Figure S2:** ACF plot comparing weekly laboratory-confirmed RSV infections in 5-14 years to 5-64 years. ACF plots measure the relationship between two time series, Xt and Yt.


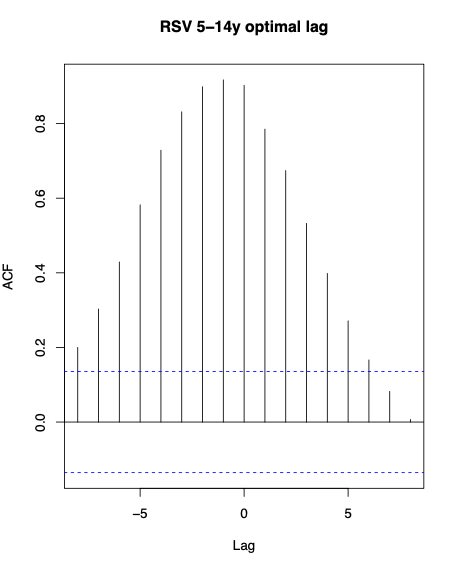


In this plot, Xt = time-series of weekly RSV infections for 5-14 years and Yt = time-series of weekly RSV infections for 5-64 years. The ACF plot demonstrates that RSV infections in 5-14 years peaks one week before RSV infections in 5-64 years (x is leading y).

**Figure S3:** Correlation matrices of laboratory-confirmed respiratory infections for age groups 0-4 years (left), 5-64 years (middle), and ≥65 years (right), with average weekly temperatures over the study period.

**
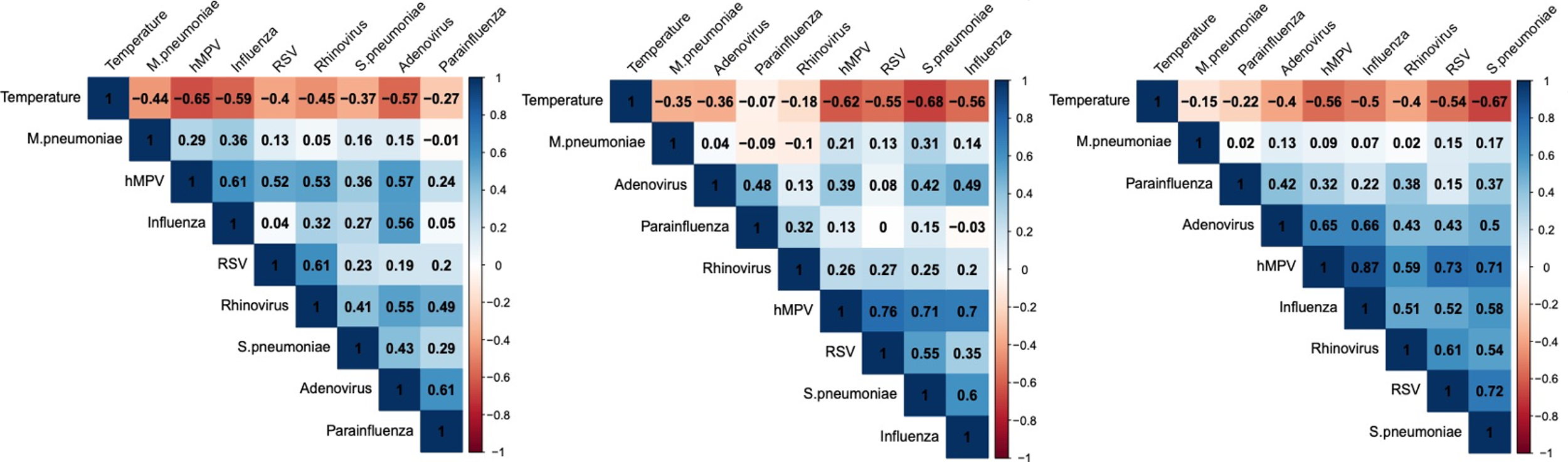
**

**2.2 Model fitting**

The best-fitting model for all ages included a moving average to smooth irregularities in pathogen counts, all practice holiday indicator variables to adjust for outliers in outcome counts, and a spline to account for unmeasured seasonal confounding (Figure S4). Models for adults ≥65 and children 5-14 years indicated potential non-linear relationships between pathogens (including influenza, RSV, hMPV, and *S. pneumoniae*) and outcomes, influenced by a few outliers in pathogen counts with high uncertainty (example in Figure S5). To address this, weekly counts of respiratory infections above the 97.5th percentile were truncated to the 97.5% value for age-specific models (Figure S5). The final set of time-varying covariates varied between age-specific models (Figure S6), with RSV, influenza, rhinovirus, and *S. pneumoniae* frequently included in models of respiratory antibiotic prescriptions. After truncation, the trend of remaining time-varying covariates was approximately linear (Figure S5); therefore, splines were removed to reduce unnecessary uncertainty.

Model residuals demonstrated reasonable normality with minimal heteroskedasticity or autocorrelation (Figure S7). Possible autocorrelation was noted in models for 0-5 months, 2-4 years and 5-14 years. However, attempts to adjust for this using Gaussian process splines^19^ suggested insufficient remaining residual trend for autocorrelation (Figure S8). Most posterior simulations of age-specific models of respiratory antibiotic prescriptions closely matched observed data (Figures S9-11), except for 0-5 months, which reflected an average of 2-4 weekly fluctuations previously noted in Scottish children.^20^ The 5-14 years model struggled to simulate a no-RSV scenario in 2018 (Figure S12). To address this, the model was adjusted by excluding 2018 data and decomposing seasonal trends into two splines to better capture long-term and repeating seasonal patterns.^21^

**Figure S4:** Development of age-specific models of respiratory antibiotic prescriptions according to Akaike Information Criterion (AIC) values.


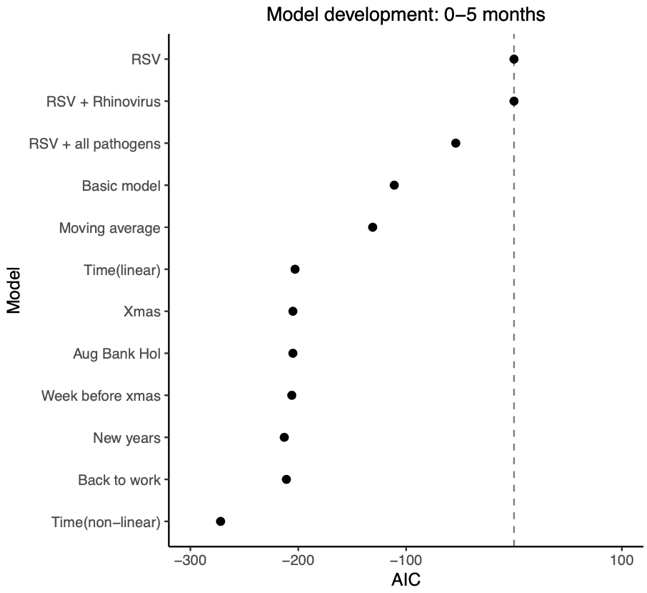

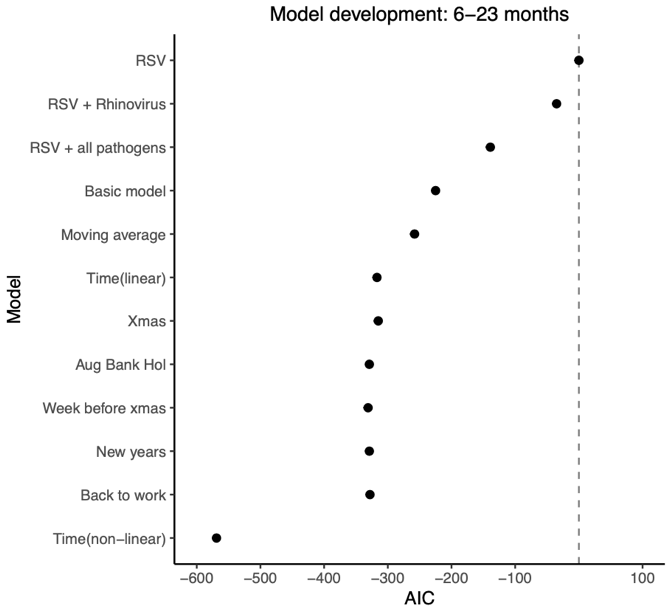

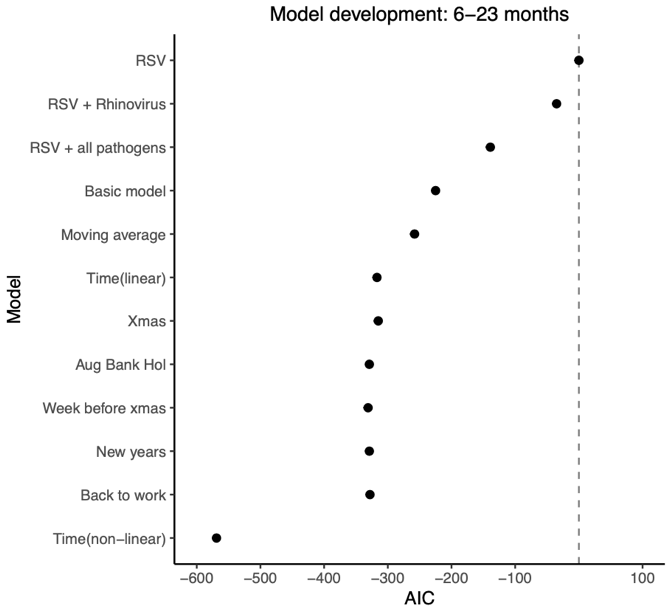

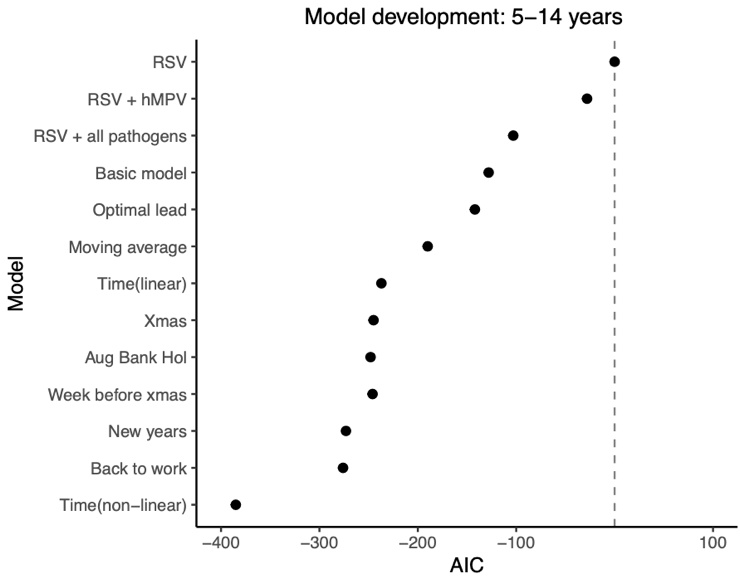

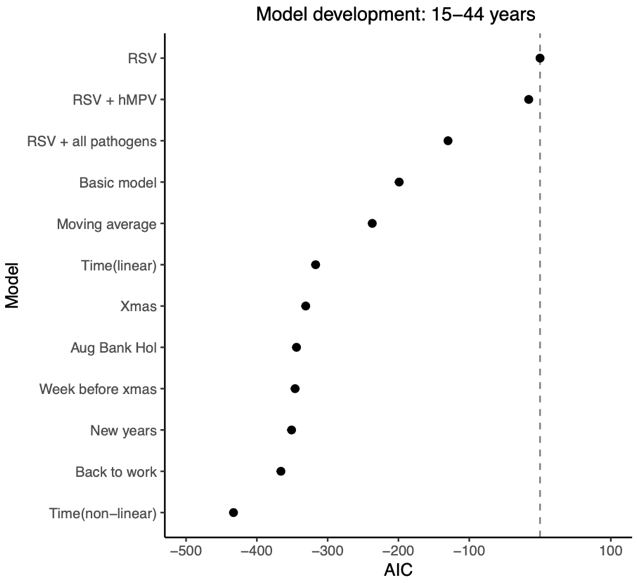

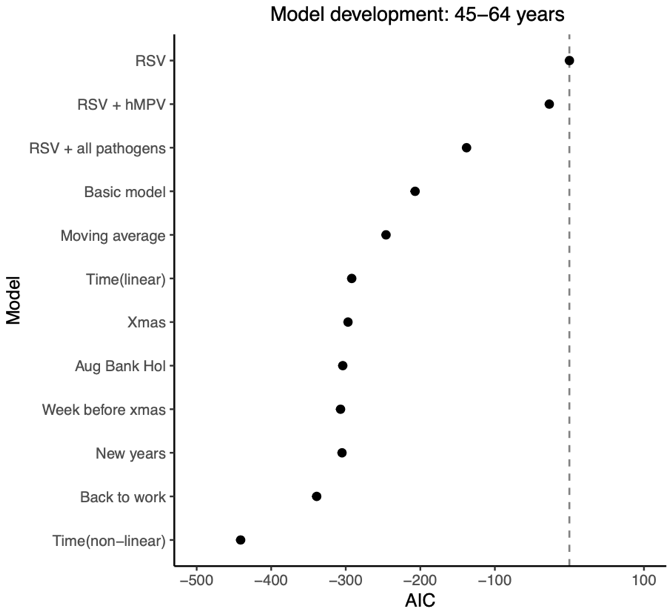

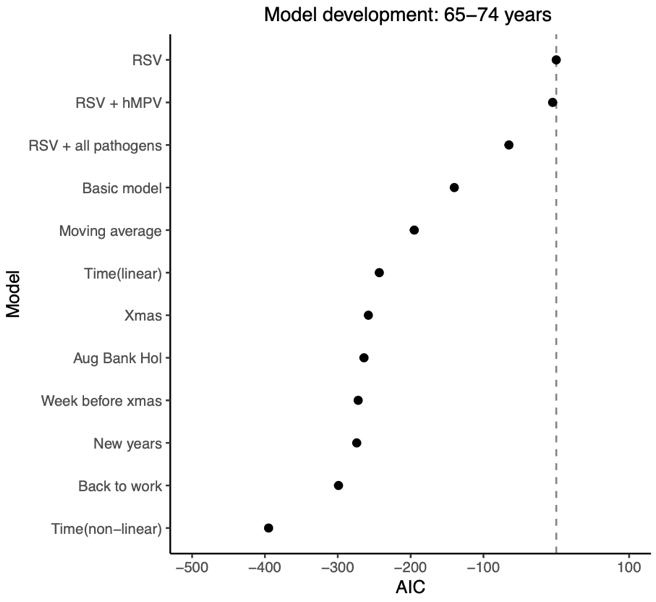

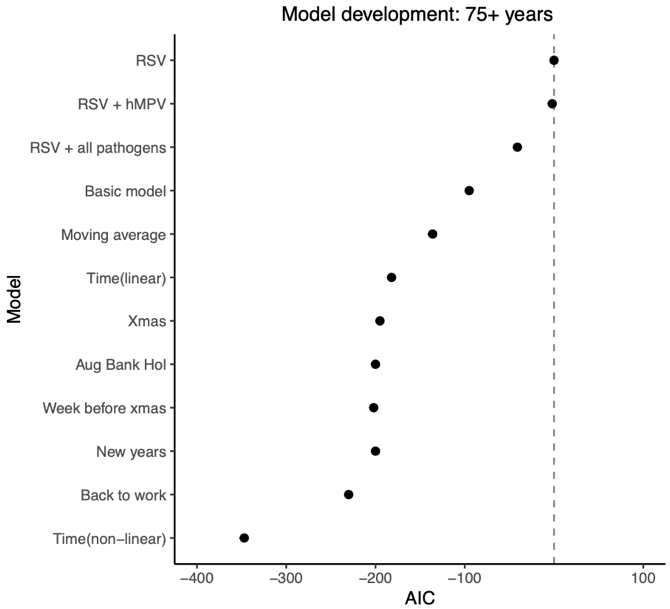


All AIC tests start with a model that includes only RSV and then evaluates the addition of the pathogen with the highest correlation with RSV in that age group (Figure S3), as well as all pathogens. The basic model comprises of all exposure variables described in the initial model equation (Equation S1). The moving average refers to the three-week moving average of laboratory-confirmed infections. Time (linear) represents a continuous variable for time (in weeks) to account for long-term linear trends in outcome counts not explained by exposures. Time (non-linear) adds a spline to control for long-term seasonal trends in outcome counts not explained by exposures. Indicator variables for practice holiday periods to adjust for outliers in antibiotic prescriptions include Xmas (Christmas holiday week), Aug Bank Hol (August Bank holiday week), New Years (week of New Year's), and back to work (week after New Year's).

Including a moving average for pathogen counts and outcome counts with a non-linear trend (spline) consistently provided the best or near-best fit across all age groups. Most indicators of practice closures had little impact on AIC for all age groups, except for the "back to work" week, which improved the model AIC for adults.

**Figure S5:** An example of partial effect plots for the ≥75 years model of respiratory antibiotic prescriptions before truncating (Top) and after truncating (Below).


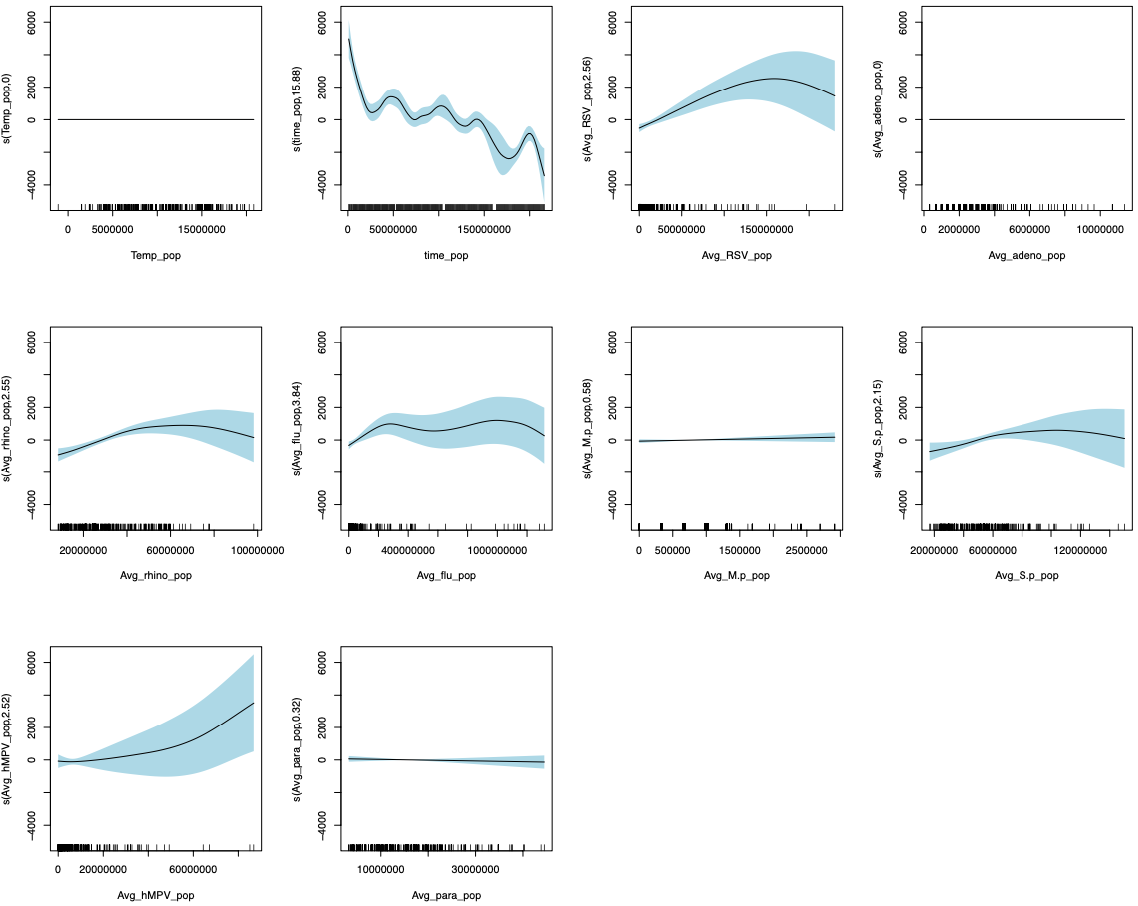


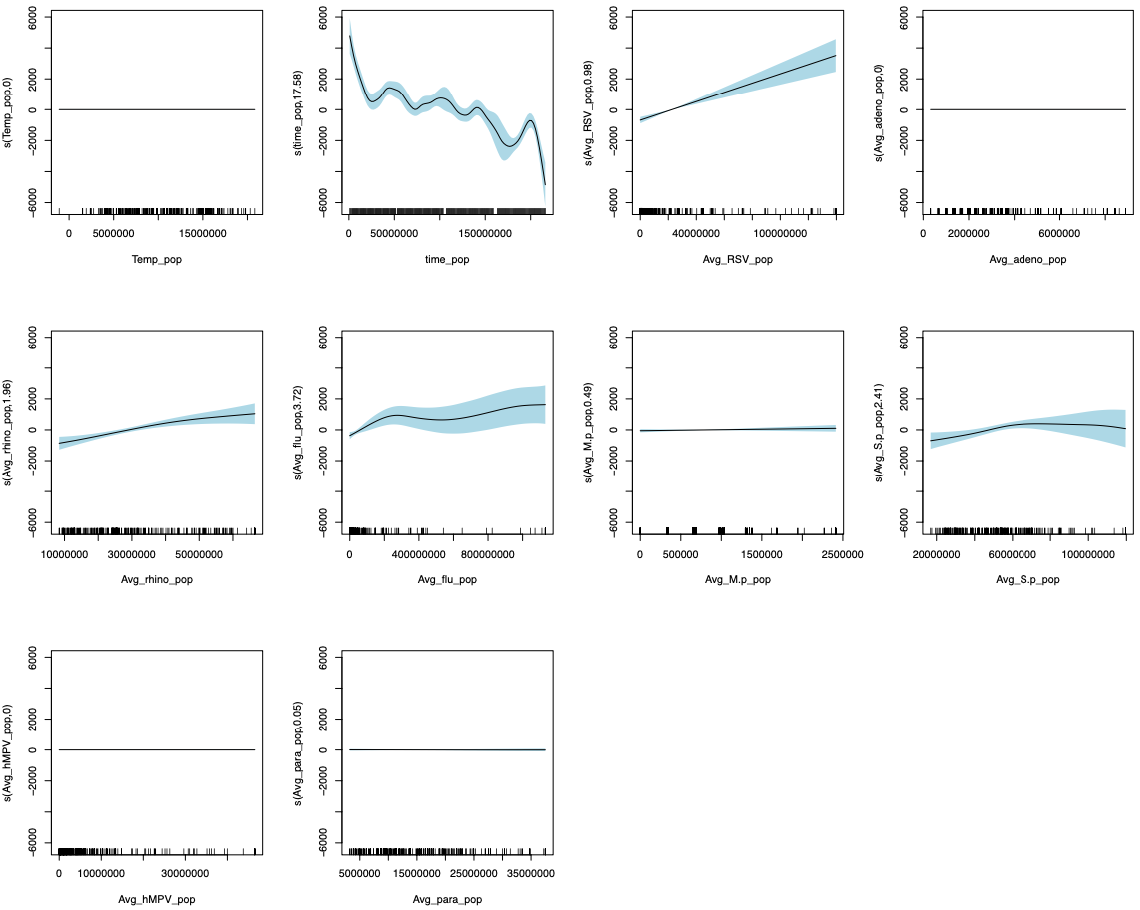


The X-axis is weekly counts of pathogens or average temperatures multiplied by the age-specific mid-year population (e.g. Avg_path_pop). The shaded blue area represents 95% confidence intervals of the predicted trend. A straight flat line (edf = 0) represents no relationship. Temp = temperature, adeno = adenovirus, rhino = rhinovirus, flu = influenza, M.p = *M. pneumoniae*, S.p = *S. pneumoniae*, para = parainfluenza.

**Figure S6:** The final sets of time-varying explanatory covariates for all separate age-specific models of outcomes demonstrated in the tables A-L below, inclusion indicated by an X.

| **A) Covariate** | **Respiratory antibiotics** | | | | | | | |
| --- | --- | --- | --- | --- | --- | --- | --- | --- |
|  | **0-5 m** | **6-23m** | **2-4 y** | **5-14 y** | **15-44y** | **45-64y** | **65-74y** | **≥75y** |
| **RSV** | x | x | x | x | x | x | x | x |
| **Influenza** | x | x | x | x | x | x | x | x |
| **Adenovirus** | x | x | x | x | x |  | x |  |
| **Rhinovirus** | x | x | x | x | x | x | x | x |
| **Parainfluenza** |  | x | x | x |  | x |  | x |
| **hMPV** | x |  | x | x |  | x |  |  |
| ***S. pneumoniae*** |  | x | x | x | x | x | x | x |
| ***M. pneumoniae*** |  | x |  | x | x | x |  | x |
| **Temperature** |  |  | x | x | x | x | x |  |

| **B) Covariate** | **Total antibiotics** | | | | | | | |
| --- | --- | --- | --- | --- | --- | --- | --- | --- |
|  | **0-5 m** | **6-23m** | **2-4 y** | **5-14 y** | **15-44y** | **45-64y** | **65-74y** | **≥75y** |
| **RSV** | x | x | x | x |  | x | x | x |
| **Influenza** |  | x | x | x |  | x |  |  |
| **Adenovirus** | x | x | x | x |  | x |  |  |
| **Rhinovirus** | x | x | x | x | x | x | x | x |
| **Parainfluenza** |  | x | x | x | x |  | x | x |
| **hMPV** |  |  | x | x |  |  |  |  |
| ***S. pneumoniae*** |  | x | x | x | x | x | x | x |
| ***M. pneumoniae*** |  | x |  | x | x | x |  | x |
| **Temperature** | x |  | x | x | x | x | x |  |

| **C) Covariate** | **Specific antibiotics potentially important for resistance** | | | | | | | |
| --- | --- | --- | --- | --- | --- | --- | --- | --- |
|  | **0-5 m** | **6-23m** | **2-4 y** | **5-14 y** | **15-44y** | **45-64y** | **65-74y** | **≥75y** |
| **RSV** | x | x | x | x |  | x | x | x |
| **Influenza** |  | x | x | x | x | x | x | x |
| **Adenovirus** | x | x | x | x | x | x | x |  |
| **Rhinovirus** | x | x | x | x | x | x | x | x |
| **Parainfluenza** |  | x | x | x | x |  | x | x |
| **hMPV** |  |  | x | x |  |  | x |  |
| ***S. pneumoniae*** |  | x | x | x | x | x | x | x |
| ***M. pneumoniae*** |  | x |  | x | x | x |  | x |
| **Temperature** | x |  | x | x | x |  |  |  |

| **D) Covariate** | **Nitrofurantoin prescriptions** | | | | | | | |
| --- | --- | --- | --- | --- | --- | --- | --- | --- |
|  | **0-5 m** | **6-23m** | **2-4 y** | **5-14 y** | **15-44y** | **45-64y** | **65-74y** | **≥75y** |
| **RSV** |  |  |  | x | x | x | x | x |
| **Influenza** |  |  |  | x | x | x | x | x |
| **Adenovirus** |  |  | x | x | x | x | x |  |
| **Rhinovirus** |  |  |  | x | x |  |  |  |
| **Parainfluenza** |  | x |  | x | x | x | x | x |
| **hMPV** |  |  |  | x | x | x | x | x |
| ***S. pneumoniae*** |  |  | x | x | x | x |  |  |
| ***M. pneumoniae*** |  |  | x | x |  |  |  | x |
| **Temperature** |  |  | x | x | x | x |  |  |

| **E) Covariate** | **Antibiotic classes 0-5m** | | | | | | | | | | |
| --- | --- | --- | --- | --- | --- | --- | --- | --- | --- | --- | --- |
|  | **Pen** | **Ceph** | **Tet** | **Amino** | **Mac** | **Clin** | **Other** | **Sulf** | **Met** | **Quin** | **UTI** |
| **RSV** | x |  |  |  | x |  |  | x |  |  |  |
| **Influenza** | x | x |  |  | x |  |  |  |  |  |  |
| **Adenovirus** | x | x |  |  | x |  |  | x |  |  |  |
| **Rhinovirus** | x |  |  |  | x |  |  |  |  |  |  |
| **Parainfluenza** |  |  |  |  |  |  |  | x |  |  |  |
| **hMPV** |  |  |  |  | x |  |  |  |  |  |  |
| ***S. pneumoniae*** |  |  |  |  |  |  |  | x |  |  |  |
| ***M. pneumoniae*** |  |  |  |  | x |  |  | x |  |  |  |
| **Temperature** |  |  |  |  | x |  |  |  |  |  |  |

| **F) Covariate** | **Antibiotic classes 6-23m** | | | | | | | | | | |
| --- | --- | --- | --- | --- | --- | --- | --- | --- | --- | --- | --- |
|  | **Pen** | **Ceph** | **Tet** | **Amino** | **Mac** | **Clin** | **Other** | **Sulf** | **Met** | **Quin** | **UTI** |
| **RSV** | x | x |  | x | x |  |  |  |  |  |  |
| **Influenza** | x | x |  | x | x |  |  |  |  | x |  |
| **Adenovirus** | x | x |  | x | x |  |  |  |  | x |  |
| **Rhinovirus** | x |  |  |  | x |  |  | x |  |  |  |
| **Parainfluenza** | x |  |  |  | x |  |  |  |  |  | x |
| **hMPV** |  |  |  |  | x |  |  |  |  |  |  |
| ***S. pneumoniae*** | x | x |  | x | x |  |  |  |  | x |  |
| ***M. pneumoniae*** | x | x |  |  |  |  |  |  |  |  |  |
| **Temperature** |  |  |  | x | x |  |  | x |  |  |  |
| **G) Covariate** | **Antibiotic classes 2-4y** | | | | | | | | | | |
|  | **Pen** | **Ceph** | **Tet** | **Amino** | **Mac** | **Clin** | **Other** | **Sulf** | **Met** | **Quin** | **UTI** |
| **RSV** | x | x |  |  | x |  | x | x |  | x |  |
| **Influenza** | x | x |  | x | x |  |  |  |  | x |  |
| **Adenovirus** | x |  |  | x | x |  |  |  |  | x | x |
| **Rhinovirus** | x | x |  | x | x |  | x | x |  |  |  |
| **Parainfluenza** | x |  |  |  | x |  |  | x |  |  |  |
| **hMPV** | x |  |  | x |  |  |  | x |  | x |  |
| ***S. pneumoniae*** | x |  |  | x | x |  |  | x | x |  | x |
| ***M. pneumoniae*** |  | x |  | x |  |  |  |  |  |  | x |
| **Temperature** | x | x |  | x |  |  | x | x |  | x | x |

| **H) Covariate** | **Antibiotic classes 5-14y** | | | | | | | | | | |
| --- | --- | --- | --- | --- | --- | --- | --- | --- | --- | --- | --- |
|  | **Pen** | **Ceph** | **Tet** | **Amino** | **Mac** | **Clin** | **Other** | **Sulf** | **Met** | **Quin** | **UTI** |
| **RSV** | x |  |  |  | x |  | x |  |  |  |  |
| **Influenza** | x | x | x | x | x |  |  | x |  |  | x |
| **Adenovirus** | x | x | x |  | x |  |  | x |  | x | x |
| **Rhinovirus** | x | x |  | x | x |  | x | x | x | x | x |
| **Parainfluenza** |  |  |  |  |  |  |  | x |  | x |  |
| **hMPV** | x |  | x |  | x |  |  | x |  |  |  |
| ***S. pneumoniae*** | x |  |  | x | x |  |  |  |  |  |  |
| ***M. pneumoniae*** | x |  | x |  |  |  |  |  |  |  | x |
| **Temperature** | x | x | x | x | x |  | x | x |  |  |  |

| **I) Covariate** | **Antibiotic classes 15-44y** | | | | | | | | | | |
| --- | --- | --- | --- | --- | --- | --- | --- | --- | --- | --- | --- |
|  | **Pen** | **Ceph** | **Tet** | **Amino** | **Mac** | **Clin** | **Other** | **Sulf** | **Met** | **Quin** | **UTI** |
| **RSV** |  |  |  | x |  |  |  |  | x |  | x |
| **Influenza** | x | x | x | x | x | x | x | x | x |  | x |
| **Adenovirus** | x | x |  | x |  |  | x |  | x | x | x |
| **Rhinovirus** | x | x | x |  | x |  |  | x | x |  | x |
| **Parainfluenza** | x | x | x |  | x | x |  | x | x | x | x |
| **hMPV** |  |  | x | x |  | x | x |  |  |  | x |
| ***S. pneumoniae*** | x | x |  | x | x | x |  | x | x | x | x |
| ***M. pneumoniae*** | x | x | x | x | x |  |  | x | x | x |  |
| **Temperature** | x | x | x |  | x |  |  | x | x | x | x |

| **J) Covariate** | **Antibiotic classes 45-64y** | | | | | | | | | | |
| --- | --- | --- | --- | --- | --- | --- | --- | --- | --- | --- | --- |
|  | **Pen** | **Ceph** | **Tet** | **Amino** | **Mac** | **Clin** | **Other** | **Sulf** | **Met** | **Quin** | **UTI** |
| **RSV** | x |  | x | x | x |  | x |  | x |  | x |
| **Influenza** | x | x | x | x | x | x | x |  | x |  | x |
| **Adenovirus** | x | x | x | x | x |  | x | x | x | x | x |
| **Rhinovirus** | x |  | x | x | x | x |  |  |  |  |  |
| **Parainfluenza** |  |  |  | x |  | x |  | x |  | x | x |
| **hMPV** |  |  | x | x |  | x |  |  |  | x | x |
| ***S. pneumoniae*** | x | x | x | x | x |  |  | x | x | x | x |
| ***M. pneumoniae*** | x | x | x | x | x |  | x | x |  | x |  |
| **Temperature** |  |  | x | x | x | x | x |  |  | x | x |

| **K) Covariate** | **Antibiotic classes 65-74y** | | | | | | | | | | |
| --- | --- | --- | --- | --- | --- | --- | --- | --- | --- | --- | --- |
|  | **Pen** | **Ceph** | **Tet** | **Amino** | **Mac** | **Clin** | **Other** | **Sulf** | **Met** | **Quin** | **UTI** |
| **RSV** | x | x | x | x | x |  | x | x | x |  | x |
| **Influenza** | x |  | x | x | x | x | x | x |  |  | x |
| **Adenovirus** | x |  |  |  | x |  | x |  | x |  |  |
| **Rhinovirus** | x | x | x |  | x | x |  |  |  |  |  |
| **Parainfluenza** | x |  | x | x |  |  |  | x | x | x | x |
| **hMPV** |  | x | x | x | x |  | x | x | x | x | x |
| ***S. pneumoniae*** | x |  | x | x | x |  |  |  | x |  |  |
| ***M. pneumoniae*** |  |  |  | x |  | x |  |  | x | x |  |
| **Temperature** | x | x | x | x |  | x |  | x |  | x |  |

| **L) Covariate** | **Antibiotic classes ≥75y** | | | | | | | | | | |
| --- | --- | --- | --- | --- | --- | --- | --- | --- | --- | --- | --- |
|  | **Pen** | **Ceph** | **Tet** | **Amino** | **Mac** | **Clin** | **Other** | **Sulf** | **Met** | **Quin** | **UTI** |
| **RSV** | x | x | x | x | x |  |  | x | x | x | x |
| **Influenza** |  |  | x | x | x |  | x |  |  |  | x |
| **Adenovirus** |  |  | x |  | x | x |  |  |  |  |  |
| **Rhinovirus** | x |  | x | x | x | x | x |  | x | x |  |
| **Parainfluenza** | x | x |  | x |  |  | x | x | x | x | x |
| **hMPV** |  | x |  | x | x |  |  | x |  | x | x |
| ***S. pneumoniae*** | x |  | x | x | x | x |  | x | x |  |  |
| ***M. pneumoniae*** | x |  | x | x |  | x |  | x | x |  | x |
| **Temperature** | x | x | x | x | x | x |  | x | x | x |  |

Tables E-L demonstrate explanatory time-changing covariates with evidence of a relationship with the outcome of interest for separate age-specific models of antibiotic classes. Class columns highlighted in red indicate that the model was not run because age-specific counts were <1,000 for the entire study period.

**Figure S7:** Evidence of residual stationarity for separate age-specific models of respiratory antibiotic prescriptions, including ACF to test for autocorrelation and quantile-quantile plots (q-q) to test for normality of residuals.


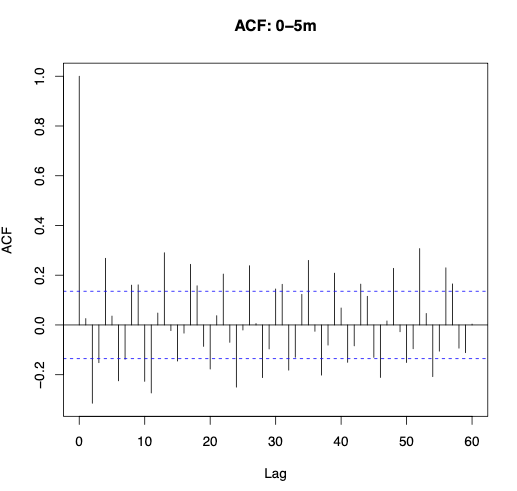

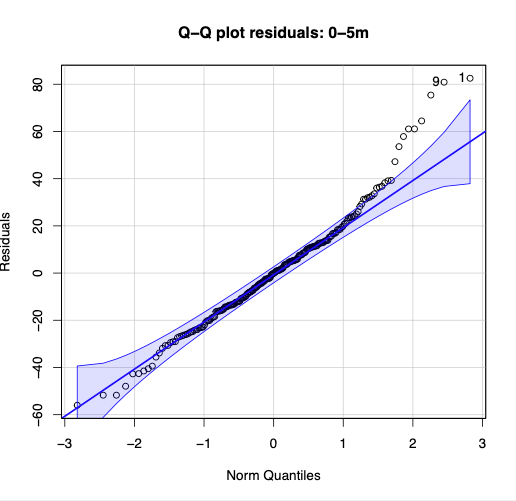


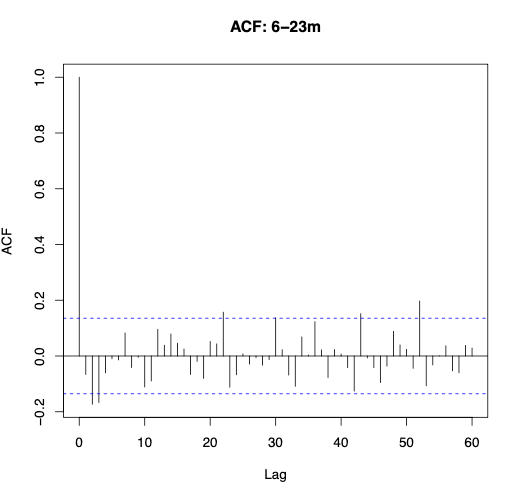

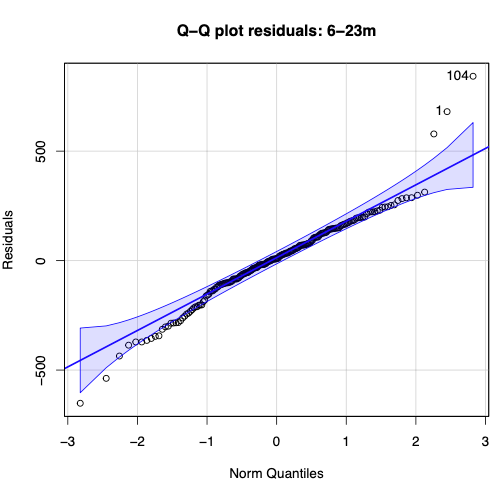

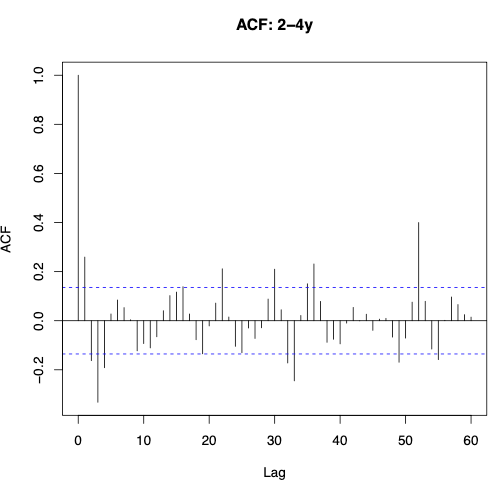

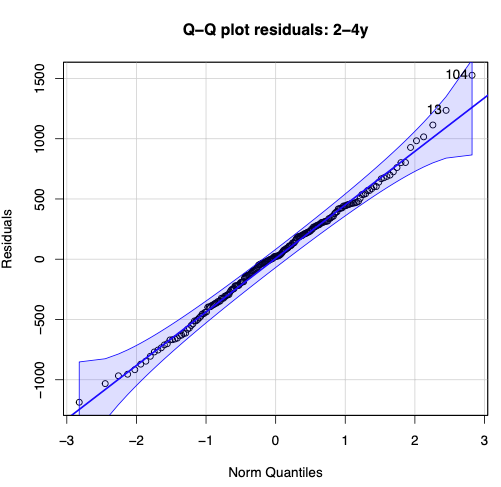

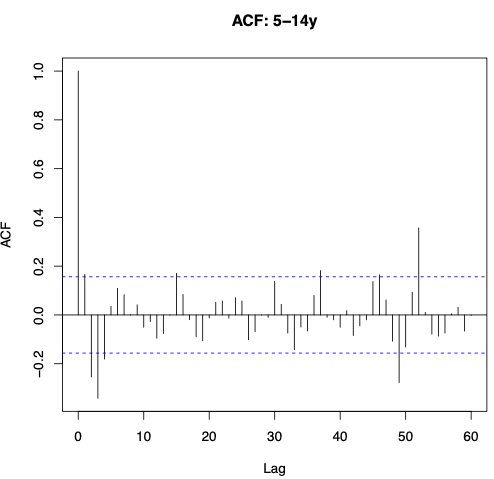

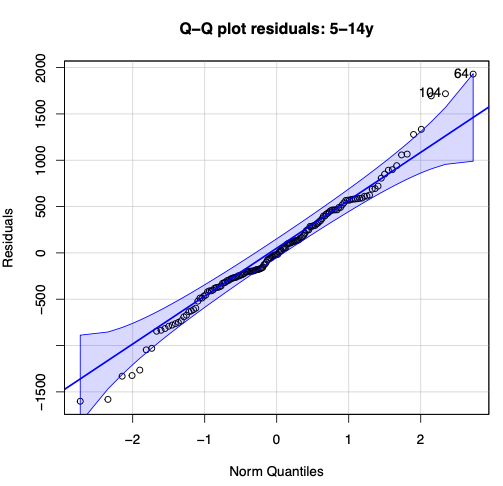
**
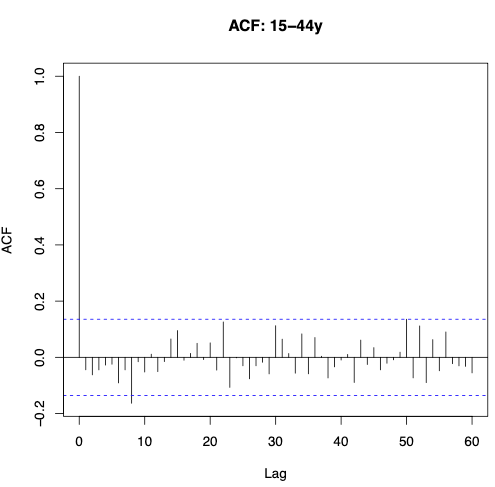

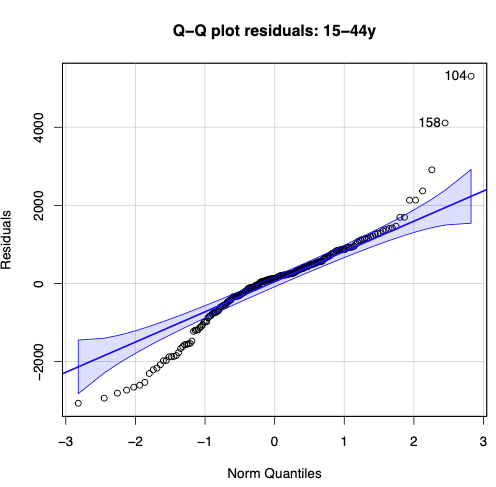

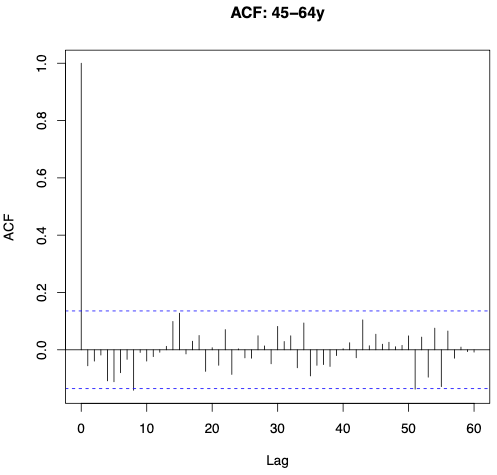

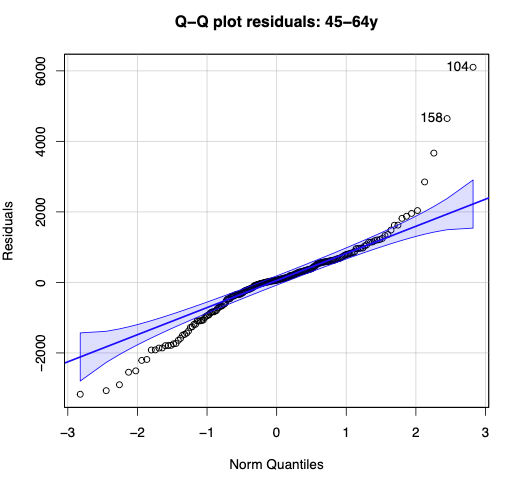

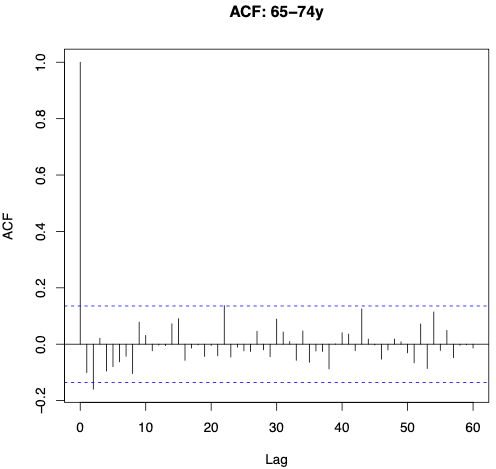

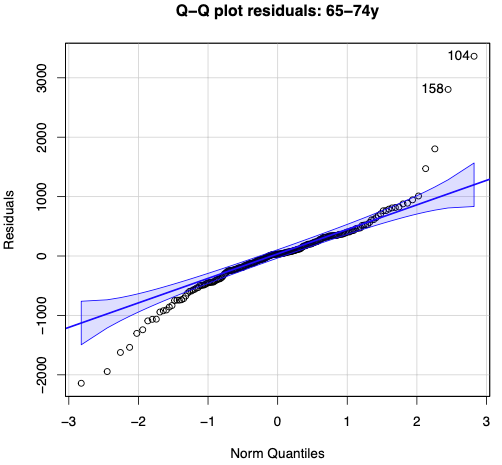

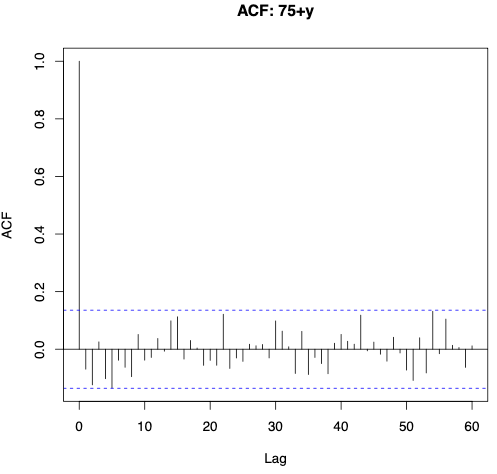

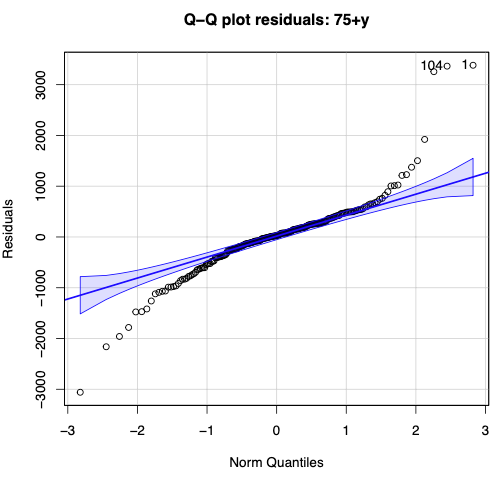
**

Overall, model residuals demonstrated reasonable normality with minimal heteroskedasticity or autocorrelation. ACF plots indicated potential autocorrelation for infants 0-5 months, children 2-4 years and 5-14 years.

**Figure S8:** Attempts of autocorrelation adjustments for models of respiratory antibiotic prescriptions in 0-5 months, 2-4 years, and 5-14 years age groups

6-23 months

0-5 months


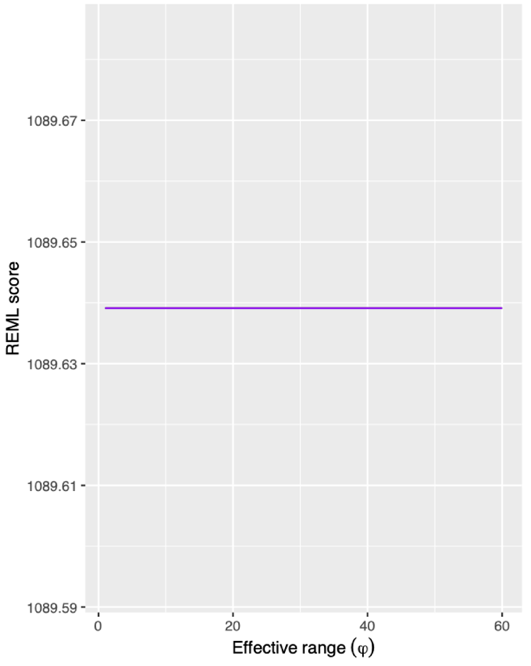

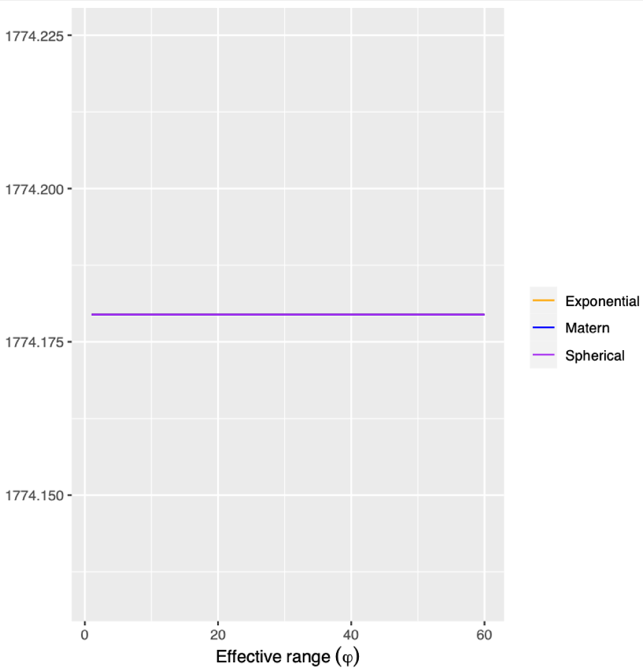


Mock data

5-14 years


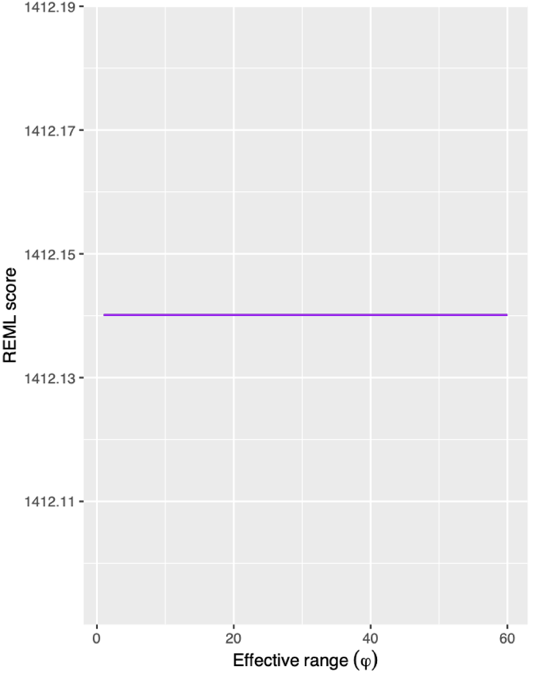

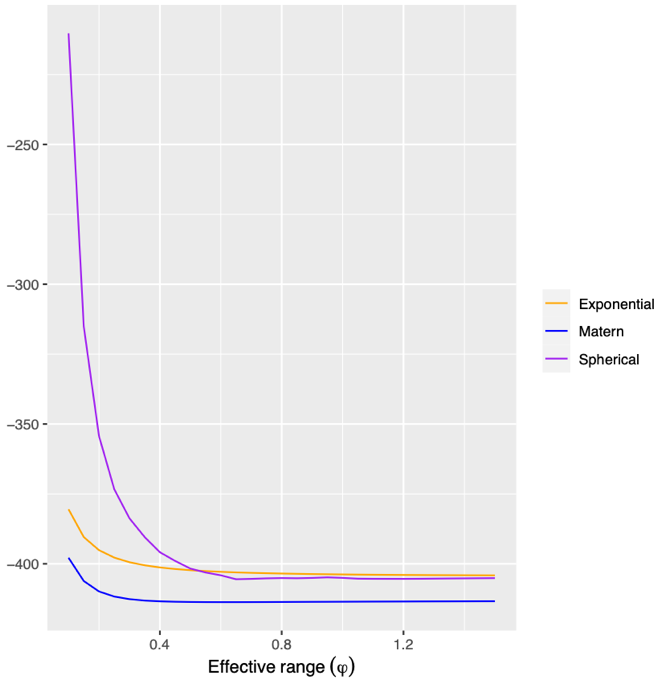


In GAMs, distinguishing between wiggly trends and autocorrelation is challenging.^19^ To adjust for autocorrelation, Gaussian process splines were applied following Simpson’s methodology.^19^ These splines use a penalised likelihood method similar to GAMS, but with an additional penalty from a correlation function. The first step involves identifying an effective range of Rho values (weekly lag) that provides the best REML score. GAMs were tested with weekly lags (1–60) using Matèrn, Squared Exponential, and Spherical correlation functions, and the REML score was extracted for each. The Rho value that minimised the REML score was identified through plotting. However, no change in REML was observed for any Rho value for all models. The Gaussian process spline method was validated with a mock dataset from stack exchange^22^ producing similar Rho values that minimised the spherical correlation function's REML score (around 0.65).

**Figure S9:** Average model posterior simulations of respiratory antibiotic prescriptions compared to observations for scenarios where RSV is present and absent for age groups between 0-4 years over the study period.


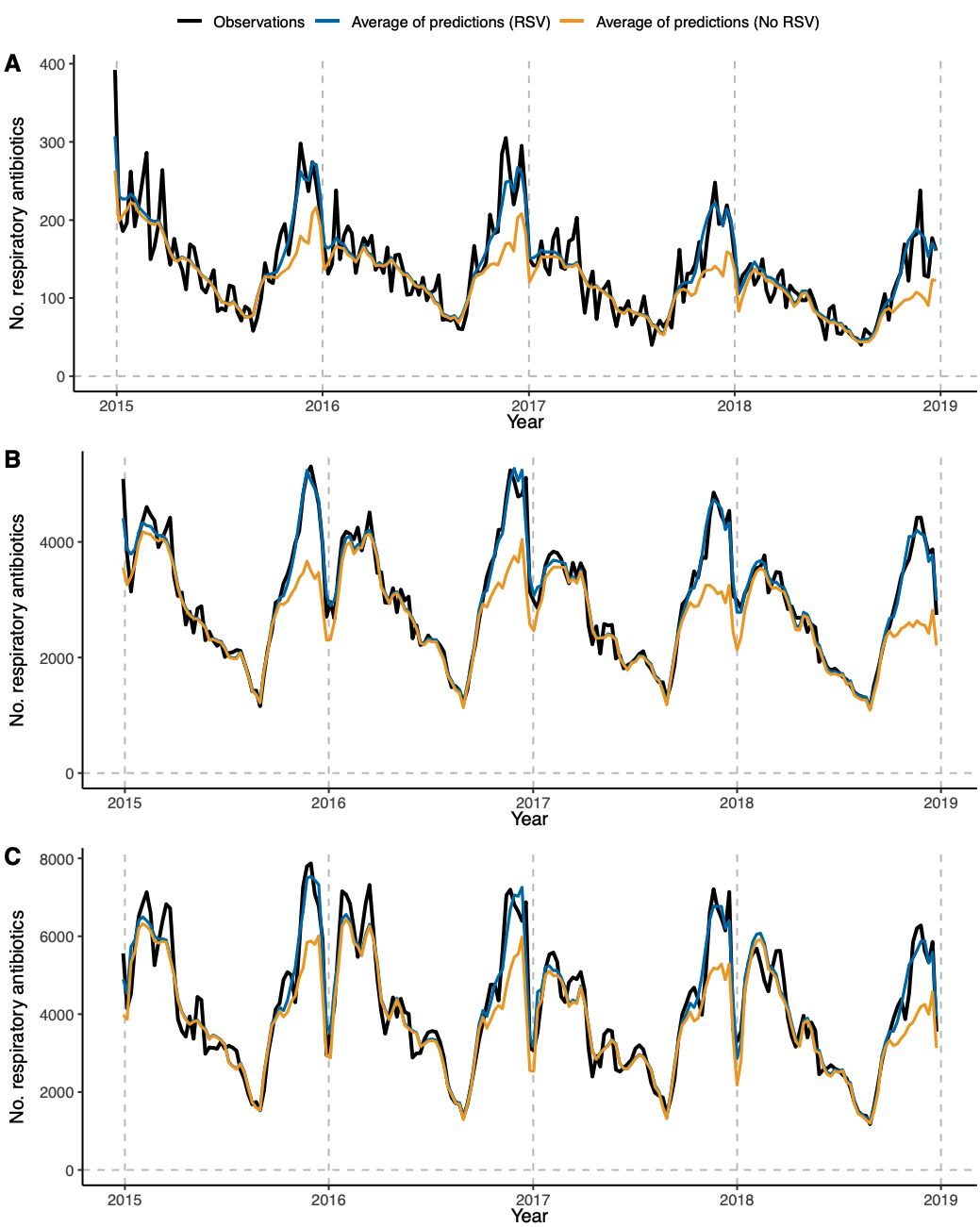


Black line = observational counts of weekly respiratory antibiotic prescriptions, blue line = average posterior simulations of weekly respiratory antibiotic prescriptions for a scenario where RSV is present, orange line = average posterior simulations of weekly respiratory antibiotic prescriptions for a scenario where RSV is absent. A = 0-5 months, B = 6-23 months, C = 2-4 years.

**Figure S10:** Average model posterior simulations of respiratory antibiotic prescriptions compared to observations for scenarios where RSV is present and absent for age groups between 5-64 years over the study period.


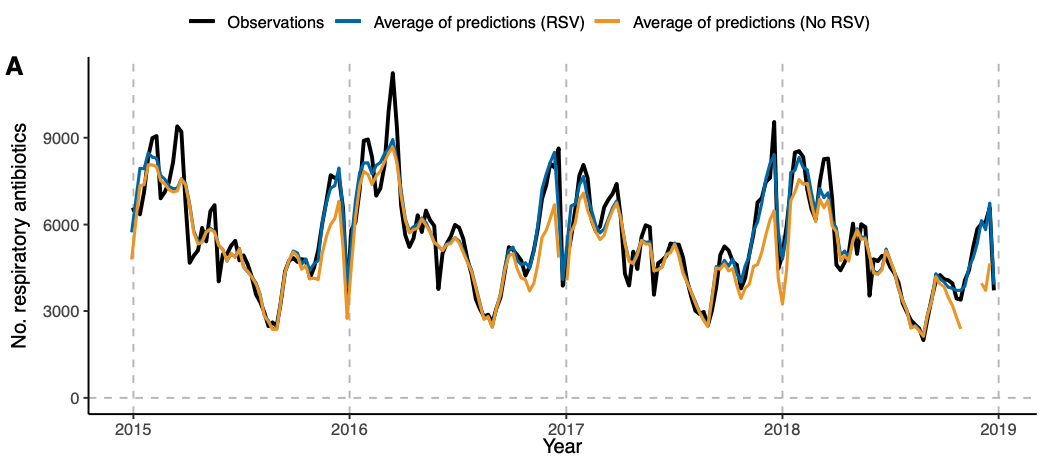

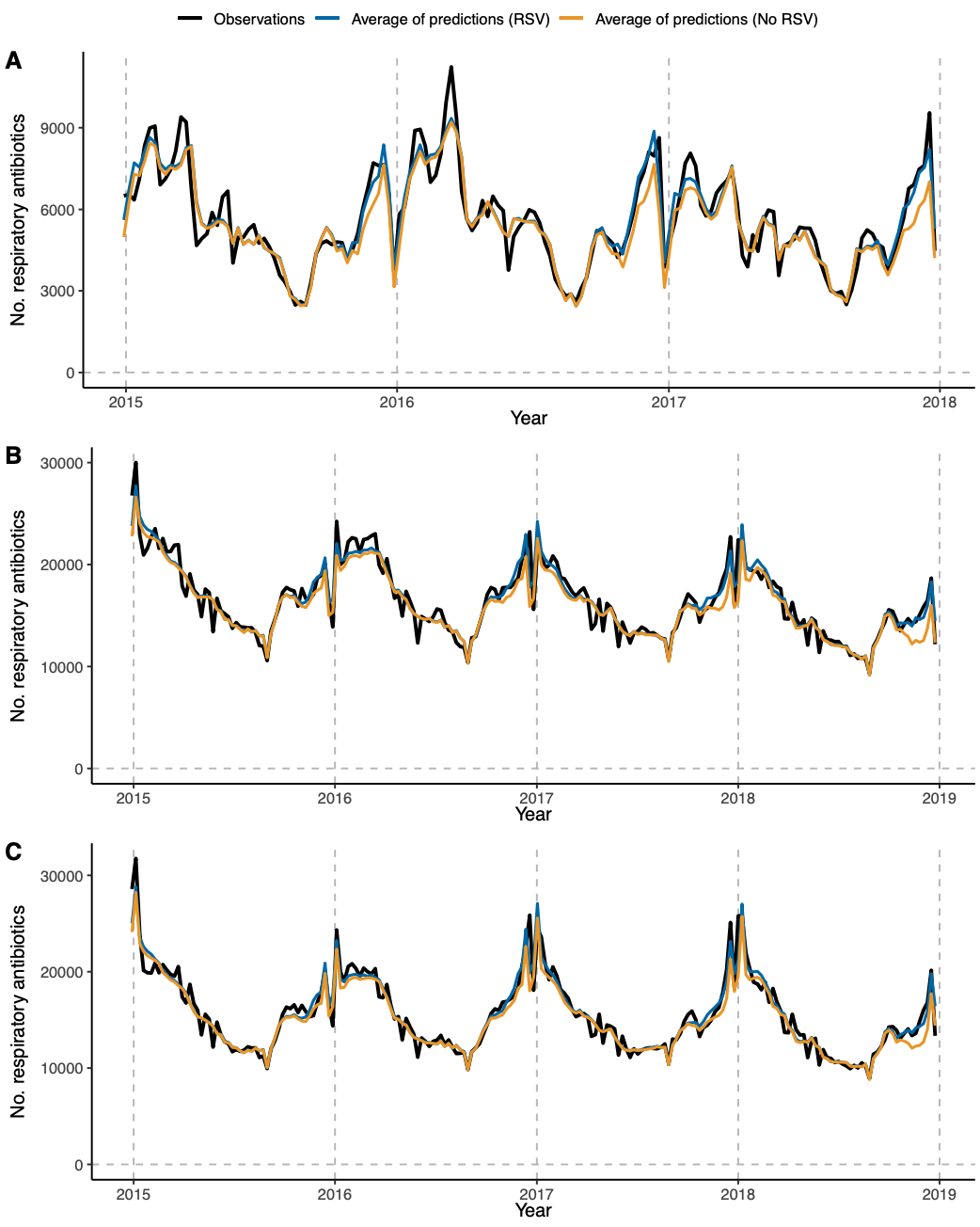


Black line = observational counts of weekly respiratory antibiotic prescriptions, blue line = average posterior simulations of weekly respiratory antibiotic prescriptions for a scenario where RSV is present, orange line = average posterior simulations of weekly respiratory antibiotic prescriptions for a scenario where RSV is absent. A = 5-14 years, B = 15-44 years, C = 45-64 years.

**Figure S11:** Average model posterior simulations of respiratory antibiotic prescriptions compared to observations for scenarios where RSV is present and absent for age groups ≥65 years over the study period.

**
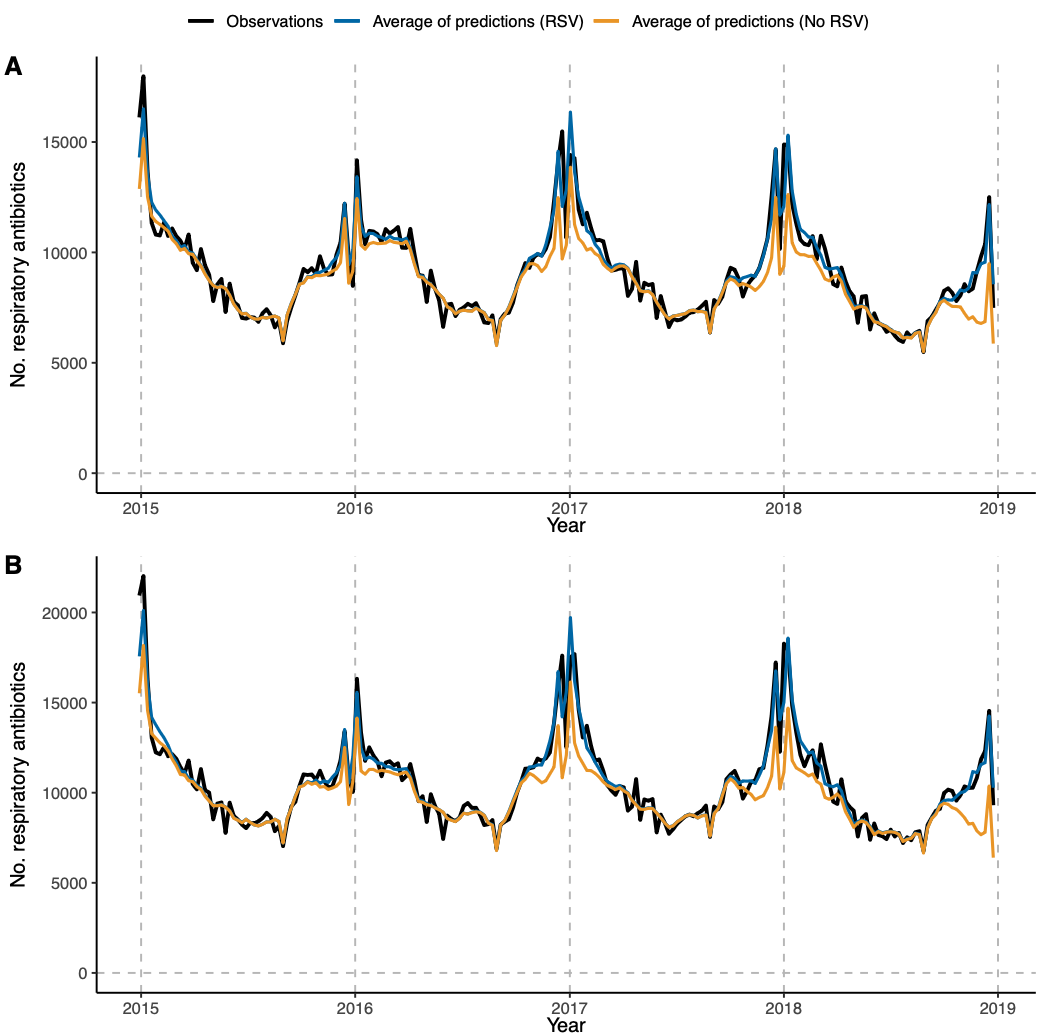
**

Black line = observational counts of weekly respiratory antibiotic prescriptions, blue line = average posterior simulations of weekly respiratory antibiotic prescriptions for a scenario where RSV is present, orange line = average posterior simulations of weekly respiratory antibiotic prescriptions for a scenario where RSV is absent. A = 65-74 years, B = ≥75 years.

**Figure S12:** Average model posterior simulations of respiratory antibiotic prescriptions compared to observations for scenarios where RSV is present and absent for alternative 5–14 years models.


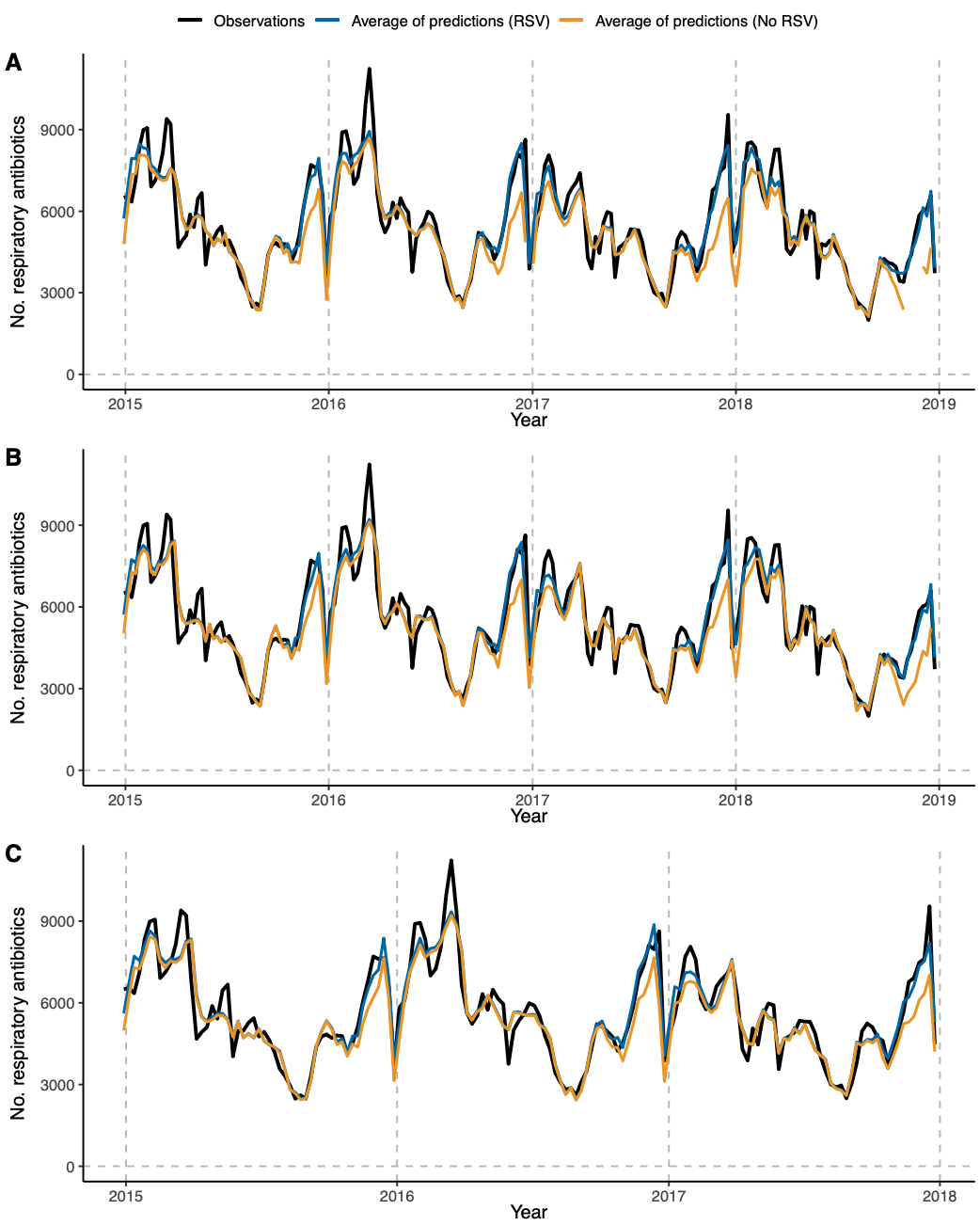


Black line = observational counts of weekly respiratory antibiotic prescriptions, blue line = average posterior simulations of weekly respiratory antibiotic prescriptions for a scenario where RSV is present, orange line = average posterior simulations of weekly respiratory antibiotic prescriptions for a scenario where RSV is absent. A = original 5–14 year model, B = 5–14 years model with seasonality trends decomposed, C = 5–14 years model with seasonality trends decomposed and counts of the final 2018 year removed.

**2.3 Secondary outcomes & negative control**

**Table S5:** Average annual RSV-attributable GP antibiotic prescriptions and respiratory antibiotic prescriptions potentially important for resistance compared to RSV-attributable GP respiratory antibiotic prescriptions from 29 December 2014 to 30 December 2018, stratified by age and based on separate age-specific models of outcomes.

|  | **Respiratory antibiotic prescriptions (primary outcome)** | | | | **Total antibiotic prescriptions** | | | | **Respiratory antibiotic prescriptions potentially important for resistance** | | | |
| --- | --- | --- | --- | --- | --- | --- | --- | --- | --- | --- | --- | --- |
| **Age** | **Prescriptions**  **(95% CrI)** | **%** | **AP % (95% CrI)** | **Rate per 100,000**  **(95% CrI)** | **Prescriptions**  **(95% CrI)** | **%** | **AP % (95% CrI)** | **Rate per 100,000**  **(95% CrI)** | **Prescriptions**  **(95% CrI)** | **%** | **AP % (95% CrI)** | **Rate per 100,000**  **(95% CrI)** |
| **0-5 m** | 6,880*  (3,211-10,167) | 1.08 | 11  (5-16) | 2,100  (984-3,100) | 7,395*  (3,554-10,944) | 1.11 | 7  (4-11) | 2,258  (1,084-3,333) | 6,997*  (3,432-10,408) | 1.06 | 8  (4-12) | 2,136  (1,050-3,180) |
| **6-23 m** | 65,535*  (45,035-86,144) | 10.24 | 10  (7-13) | 6,580  (4,522-8,651) | 66,844*  (44,408-88,939) | 10.06 | 8  (6-11) | 6,711  (4,462-8,933) | 66,524*  (43,206-89,542) | 10.10 | 9  (6-12) | 6,679  (4,346-8,994) |
| **2-4 y** | 72,500  (29,222-119,821) | 11.33 | 8  (3-13) | 3,497  (1,405-5,782) | 75,227  (25,594-126,977) | 11.33 | 6  (2-10) | 3,628  (1,250-6,132) | 74,221  (24,484-125,501) | 11.27 | 7  (2-11) | 3,580  (1,179-6,054) |
| **5-14 y #** | 55,860  (-41,759-156,160) | 8.73 | 4  (-3-12) | 857  (-637-2,397) | 62,369  (-74,815-193,426) | 9.39 | 3  (-4-9) | 956  (-1,149-2,966) | 61,682  (-59,192-172,309) | 9.36 | 4  (-3-10) | 946  (-902-2,649) |
| **15-44 y** | 95,554  (6,792-185,445) | 14.93 | 3  (0-5) | 447  (31-868) | - | - | - | - | - | - | - | - |
| **45-64 y** | 86,608  (-16,608-191,877) | 13.53 | 2  (0-6) | 612  (-117-1,357) | 136,809  (3,015-273,393) | 20.60 | 2  (0-4) | 966  (18-1,934) | 148,091  (45,216-253,682) | 22.48 | 3  (1-5) | 1,046  (319-1,790) |
| **65-74 y** | 107,893  (60,407-158,507) | 16.86 | 5  (3-7) | 1,972  (1,104-2,901) | 123,365  (34,494-212,128) | 18.57 | 3  (1-4) | 2,255  (608-3,877) | 138,206  (65,507-207,870) | 20.98 | 4  (2-6) | 2,526  (1,185-3,804) |
| **≥75 y** | 149,078  (93,733-206,045) | 23.30 | 6  (4-8) | 3,279  (2,050-4,532) | 192,246  (89,342-300,243) | 28.94 | 3  (1-5) | 4,229  (1,962-6,617) | 163,106  (85,334-246,094) | 24.76 | 4  (2-6) | 3,588  (1,872-5,415) |

AP = Attributable Proportion, % = age-specific proportion, CrI = credible interval, m = months, y = years. *= Prescriptions are estimated assuming English mid-year populations are equally distributed by month of age as ONS population estimates are only provided by year of age, - = The model was not run as age-specific counts of antibiotics demonstrated no relationship with confirmed RSV infections, # = Average annual RSV-attributable prescriptions for 5-14 years were estimated from 2015 to 2017 and assumed to apply to 2018, as 2018 data was excluded for this group (see section 2.2 model fitting).

**Table S6:** Age-specific class proportion % of RSV-attributable GP antibiotic prescriptions and respiratory antibiotic prescriptions, based on separate age-specific models of antibiotic classes.

| **Class proportion of age-specific RSV-attributable GP antibiotic prescriptions %** | | | | | | | | | |
| --- | --- | --- | --- | --- | --- | --- | --- | --- | --- |
| **Class** | **0-5 m** | **6-23 m** | **2-4 y** | **5-14 y** | **15-44 y** | **45-64 y** | **65-74 y** | **≥ 75 y** |  |
| **PEN** | 100.0 | 90.4 | 84.0 |  |  | 65.4 | 45.2 | 48.9 |  |
| **CEPH+** |  |  | 1.0 |  |  |  | 2.5 | 3.4 |  |
| **TET** |  |  |  |  |  | 21.3 | 23.5 | 19.0 |  |
| **AMINO** |  |  |  |  |  |  | 5.0 | 3.0 |  |
| **MAC** |  | 9.6 | 15.0 |  |  | 13.3 | 17.7 | 15.0 |  |
| **CLI+** |  |  |  |  |  |  |  |  |  |
| **OTHER** |  |  |  |  |  |  | 0.6 |  |  |
| **SULF+** |  |  |  |  |  |  | 5.5 | 8.7 |  |
| **MTZ+** |  |  |  |  |  |  |  |  |  |
| **QUIN** |  |  |  |  |  |  |  | 2.0 |  |
| **Class proportion of age-specific RSV-attributable GP respiratory antibiotic prescriptions %** | | | | | | | | | |
| **PEN** | 100.0 | 90.4 | 84.9 |  |  | 65.4 | 52.4 | 59.0 |  |
| **TET** |  |  |  |  |  | 21.3 | 27.2 | 22.9 |  |
| **MAC** |  | 9.6 | 15.1 |  |  | 13.3 | 20.4 | 18.1 |  |

m = months, y = years, PEN = Penicillin's, CEPH+ = Cephalosporins & other beta lactams, TET = Tetracyclines, AMINO = Aminoglycosides, MAC = Macrolides, CLI+ = Clindamycin & Lincomycin, OTHER = Other antibacterials, SULF+ = Sulfonamides & Trimethoprim, MTZ+ = Metronidazole, Tinidazole & Ornidazole, QUIN = Quinolones. UTI antibiotics were excluded from class proportion estimates of RSV attributable antibiotic prescriptions as these are assumed to not be prescribed for RSV infections. Respiratory antibiotic prescriptions in this table included antibiotic classes recommended for treating RTIs namely; penicillins, macrolides and tetracyclines.^2–8^

**Table S7:** Average annual RSV-attributable GP nitrofurantoin prescriptions from 29 December 2014 to 30 December 2018, stratified by age and based on separate age-specific models of nitrofurantoin prescriptions.

| **Nitrofurantoin prescriptions (negative control)** | | | | |
| --- | --- | --- | --- | --- |
| **Age** | **Prescriptions**  **(95% CrI)** | **%** | **Attributable proportion % (95% CrI)** | **Rate per 100,000**  **(95% CrI)** |
| **0-5 m** | - | - | - | - |
| **6-23 m** | - | - | - | - |
| **2-4 y** | -33 (-324-266) | NA | -1 (-8-6) | -2 (-16-13) |
| **5-14 y** | - | - | - | - |
| **15-44 y** | 24,344 (6,588-45,611) | 32.0 | 4 (1-7) | 144 (31-214) |
| **45-64 y** | 18,232 (1,296-35,474) | 24.0 | 3 (0-5) | 129 (9-251) |
| **65-74 y** | 12,069 (1,055-22,151) | 15.9 | 2 (0-4) | 221 (18-404) |
| **≥75 y** | 21,380 (5,083-37,282) | 28.1 | 3 (1-4) | 470 (110-817) |

% = age-specific proportion, CrI = credible interval, m = months, y = years, - = The model was not run because age-specific counts of nitrofurantoin prescriptions were <1,000 during the study period or demonstrated no relationship with confirmed RSV infections. N/A = negative result not included in proportion estimate.

**Figure S13**: Posterior simulations of weekly nitrofurantoin prescriptions for 15-44 years (top) and ≥75 years (bottom) for scenarios where RSV is present and absent from 2015 to 2018.

**Table S8**: Average annual RSV-attributable GP respiratory antibiotic prescriptions with removal of highly correlated pathogens (middle) and removal of all pathogens except for RSV and Influenza (right) compared to primary outcome from 29 December 2014 to 30 December 2018, stratified by age and based on separate age-specific models of outcomes.

|  | **Primary outcome** | | | **Removal of pathogens highly correlated with RSV** | | | **Only controlling for RSV and Influenza** | | |
| --- | --- | --- | --- | --- | --- | --- | --- | --- | --- |
| **Age** | **Prescriptions**  **(95% CrI)** | **AP %**  **(95% CrI)** | **Rate per 100,000**  **(95% CrI)** | **Prescriptions**  **(95% CrI)** | **AP %**  **(95% CrI)** | **Rate per 100,000**  **(95% CrI)** | **Prescriptions**  **(95% CrI)** | **AP %**  **(95% CrI)** | **Rate per 100,000**  **(95% CrI)** |
| **0-5 m** | 6,880*  (3,211-10,167) | 11  (5-16) | 2,100  (984-3,100) |  |  |  | 9,184*  (5,819-12,671) | 15  (9-21) | 2,805  (1,781-3,870) |
| **6-23 m** | 65,535*  (45,035-86,144) | 10  (7-13) | 6,580  (4,522-8,651) |  |  |  | 115,315*  (80,856-150,746) | 18  (12-23) | 11,578  (8,118-15,132) |
| **2-4 y** | 72,500  (29,222-119,821) | 8  (3-13) | 3,497  (1,405-5,782) |  |  |  | 210,956« | 22 | 10,175« |
| **5-14 y #** | 55,860  (-41,759-156,160) | 4  (-3-12) | 857  (-637-2,397) | **-** | **-** | - | 89,828« | 7 | 1,378« |
| **15-44 y** | 95,554  (6,792-185,445) | 3  (0-5) | 447  (31-868) | 95,554  (6,792-185,445) | 3  (0-5) | 447  (31-868) | 26,442  (-99,224-141,995) | 1  (-3-4) | 124  (-465-665) |
| **45-64 y** | 86,608  (-16,608-191,877) | 2  (0-6) | 612  (-117-1,357) | 121,570  (27,099-214,454) | 3  (1-6) | 859  (191-1,515) | 84,469  (-27,732-193,481) | 2  (-1-6) | 597  (-200-1,367) |
| **65-74 y** | 107,893  (60,407-158,507) | 5  (3-7) | 1,972  (1,104-2,901) | 138,265  (89,746-186,255) | 6  (4-8) | 2,527  (1,635-3,410) | 134,198  (72,643-193,903) | 6  (3-9) | 2,453  (1,320-3,541) |
| **≥75 y** | 149,078  (93,733-206,045) | 6  (4-8) | 3,279  (2,050-4,532) | 175,083  (119,232-232,918) | 7  (5-9) | 3,852  (2,617-5,125) | 167,780  (96,502-235,420) | 7  (4-10) | 3,691  (2,119-5,188) |

AP =Attributable proportion, CrI = credible interval, m = months, y = years. – = model struggled to run, # = Average annual RSV-attributable prescriptions for 5-14 years were estimated from 2015 to 2017 and assumed to apply to 2018, as 2018 data was excluded for this group (see model fitting). *= Prescriptions are estimated assuming English mid-year populations are equally distributed by month of age as ONS population estimates are only provided by year of age, « = Only average simulations are provided by the predict function as 1000 simulations couldn’t be produced using PostSim.

**References**

1. OpenPrescribing.net, Bennett Institute for Applied Data Science, University of Oxford. *5.1: Antibacterial Drugs*. 2025 https://openprescribing.net/bnf/0501/

2. Dolk FCK, Pouwels KB, Smith DRM, *et al*. Antibiotics in primary care in England: which antibiotics are prescribed and for which conditions? *J Antimicrob Chemother* 2018; **73**: ii2–10.

3. National Institute for Health and Care Excellence (NICE). *Otitis media (acute): antimicrobial prescribing. NICE guideline [NG91].* 2018. https://www.nice.org.uk/guidance/ng91

4. National Institute for Health and Care Excellence (NICE). *Cough (acute): antimicrobial prescribing. NICE guideline [NG120].* 2019. https://www.nice.org.uk/guidance/ng120

5. National Institute for Health and Care Excellence (NICE). *Sore throat (acute): antimicrobial prescribing. NICE guideline [NG84].* 2018. https://www.nice.org.uk/guidance/ng84

6. National Institute for Health and Care Excellence (NICE). *Sinusitis (acute): antimicrobial prescribing. NICE guideline [NG79].* 2017. https://www.nice.org.uk/guidance/ng79

7. National Institute for Health and Care Excellence (NICE). *Pneumonia (community-acquired): antimicrobial prescribing. NICE guideline [NG138].* 2019. https://www.nice.org.uk/guidance/ng138/chapter/recommendations#choice-of-antibiotic

8. National Institute for Health and Care Excellence (NICE). *Bronchiolitis in children: diagnosis and management. NICE guideline [NG9].* 2015. https://www.nice.org.uk/guidance/ng9

9. Tedijanto C, Olesen SW, Grad YH, *et al.* Estimating the proportion of bystander selection for antibiotic resistance among potentially pathogenic bacterial flora. *Proc Natl Acad Sci U S A* 2018; **115**: E11988–95.

10. National Institute for Health and Care Excellence (NICE). *Urinary tract infection (lower): antimicrobial prescribing. NICE guideline [NG109].* 2018. https://www.nice.org.uk/guidance/ng109

11. Public Health England. *English surveillance programme for antimicrobial utilisation and resistance (ESPAUR): Report 2018-2019*. https://webarchive.nationalarchives.gov.uk/ukgwa/20200806045257mp_/https://assets.publishing.service.gov.uk/government/uploads/system/uploads/attachment_data/file/843129/English_Surveillance_Programme_for_Antimicrobial_Utilisation_and_Resistance_2019.pdf

12. Public Health England. *English surveillance programme for antimicrobial utilisation and resistance (ESPAUR): Report 2019 to 2020*. https://webarchive.nationalarchives.gov.uk/ukgwa/20211022024510mp_/https://assets.publishing.service.gov.uk/government/uploads/system/uploads/attachment_data/file/936199/ESPAUR_Report_2019-20.pdf

13. Pouwels KB, Freeman R, Muller-Pebody B, *et al.* Association between use of different antibiotics and trimethoprim resistance: Going beyond the obvious crude association. *J Antimicrob Chemother* 2018; **73**: 1700–7.

14. Pouwels KB, Muller-Pebody B, Smieszek T, *et al.* Selection and co-selection of antibiotic resistances among Escherichia coli by antibiotic use in primary care: An ecological analysis. *PloS One* 2019; **14**: e0218134.

15. Weber SG, Gold HS, Hooper DC, *et al.* Fluoroquinolones and the Risk for Methicillin-resistant Staphylococcus aureus in Hospitalized Patients. *Emerg Infect Dis* 2003; **9**: 1415–22.

16. Pouwels KB, Batra R, Patel A, *et al.* Will co-trimoxazole resistance rates ever go down? Resistance rates remain high despite decades of reduced co-trimoxazole consumption. *J Glob Antimicrob Resis****t*** 2017; **11**: 71–4.

17. Office for National Statistics (ONS). 2021. Estimates of the population for the UK, England and Wales, Scotland and Northern Ireland. [Dataset]. https://www.ons.gov.uk/peoplepopulationandcommunity/populationandmigration/populationestimates/datasets/populationestimatesforukenglandandwalesscotlandandnorthernireland

18. WHO Essential Medicines Team. *The WHO AWaRe (Access, Watch, Reserve) antibiotic book*. Geneva: World Health Organization. https://www.who.int/publications/i/item/9789240062382

19. Simpson GL. Modelling Palaeoecological Time Series Using Generalised Additive Models. *Frontiers in Ecology and Evolution* 2018; **6**.

20. Fitzpatrick T, Malcolm W, McMenamin J, *et al.* Community-based antibiotic prescribing attributable to respiratory syncytial virus and other common respiratory viruses in young children: a population-based time series study of scottish children. *Clin Infect Dis* 2020; **72:** 2144-2153

21. Simpson G. *Modelling seasonal data with GAMs.* From the Bottom of the Heap. https://www.fromthebottomoftheheap.net/2014/05/09/modelling-seasonal-data-with-gam/

22. Simpson G. *Answer to ‘Gaussian Process smooths in mgcv: choosing between spherical and exponential covariance functions’.* Cross Validated. https://stats.stackexchange.com/a/323461
